# Supplementary material for: Pluripotent stem cell–derived corneal endothelial cells as an alternative to donor corneal endothelium in keratoplasty
Source: Stem Cell Reports. 2021 Aug 5;16(9):2320–35. doi: 10.1016/j.stemcr.2021.07.008 (PMC8452521; doi:10.1016/j.stemcr.2021.07.008)
Supplement: Document S1. Supplemental experimental procedures, Figures S1–S7, and Tables S1 and S2 [file mmc1.pdf]

Stem Cell Reports, Volume *16*

Supplemental Information

Pluripotent stem cell–derived corneal endothelial cells as an alternative to donor corneal endothelium in keratoplasty

Muhammad Ali, Shahid Y. Khan, John D. Gottsch, Eric K. Hutchinson, Aisha Khan, and S. Amer Riazuddin

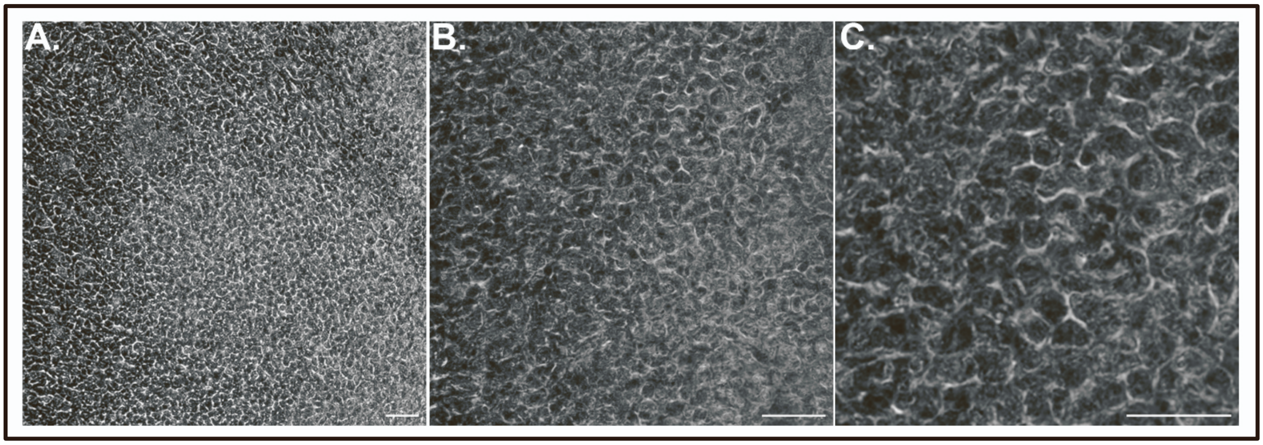

Supplementary Figure 1. Phase-contrast microscopy of human embryonic stem cell-derived corneal endothelial cells

The images of cells at differentiation day 20 are captured at (A) 10x, (B) 20x, and (C) 40x magnifications. Scale bars, 50 μm.

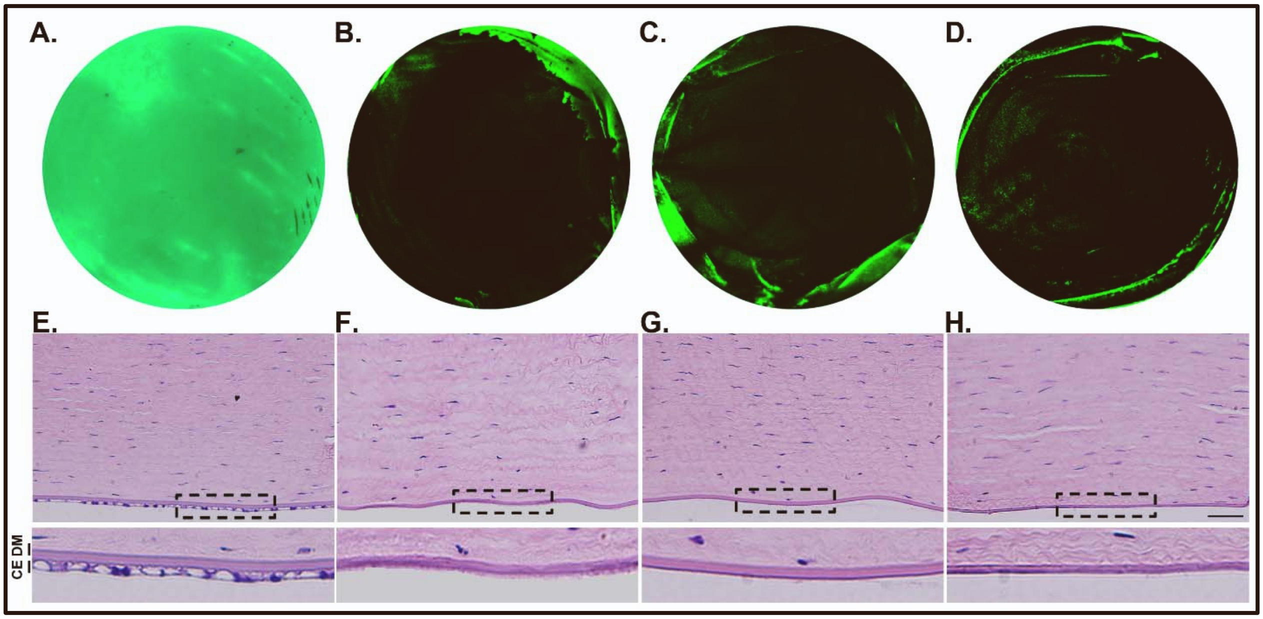

Supplementary Figure 2. Evaluation of the removal of corneal endothelium (CE) in rabbits by Calcein-AM and hematoxylin and eosin (H&E) staining

(A) An untreated control rabbit eye illustrating Calcein-AM stained intact CE. (B-D) The absence of staining confirms that the scrapped area is devoid of corneal endothelial cells (CECs) following the mechanical scraping of the CE. (E) H&E stained untreated control rabbit eye with an intact CE adherent to the Descemet's membrane (DM). (F-H) The scrapped area is devoid of CECs with an intact DM following the mechanical scraping of CE. The boxed areas are enlarged and shown in panels below. The images are captured at 20x magnification. Scale bar, 50 μm.

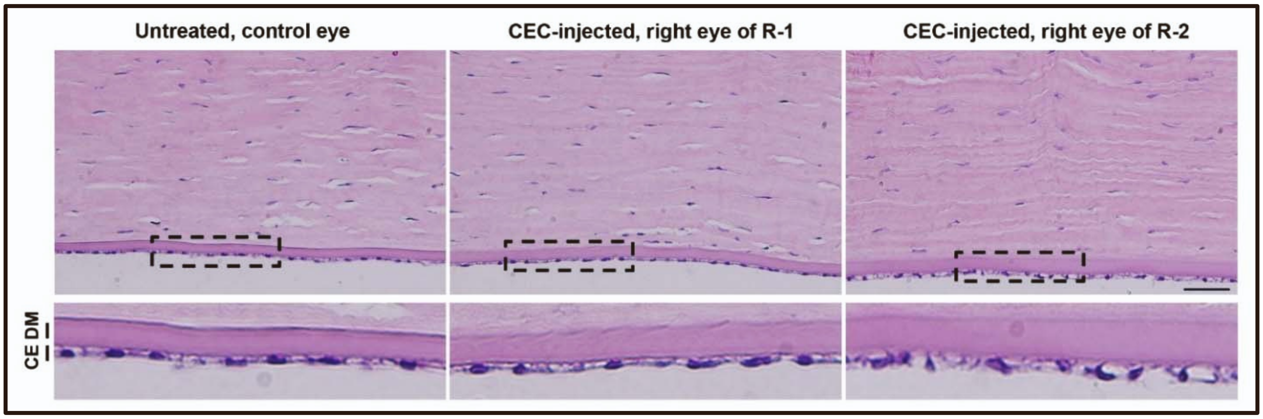

Supplementary Figure 3. Hematoxylin and eosin (H&E) staining of cryopreserved human embryonic stem cell (hESC)-derived corneal endothelial cell (CEC) injected rabbit corneas

Rabbit corneas of untreated control eye and cryopreserved hESC-derived CEC injected right eyes of R-1 and R-2. The rabbits (R-1, and R-2) represent the injection model where hESC-derived CECs are injected immediately after the removal of the central corneal endothelium. The boxed areas are enlarged and shown in panels below. The images are captured at 20x magnification. Scale bar, 50 μm.

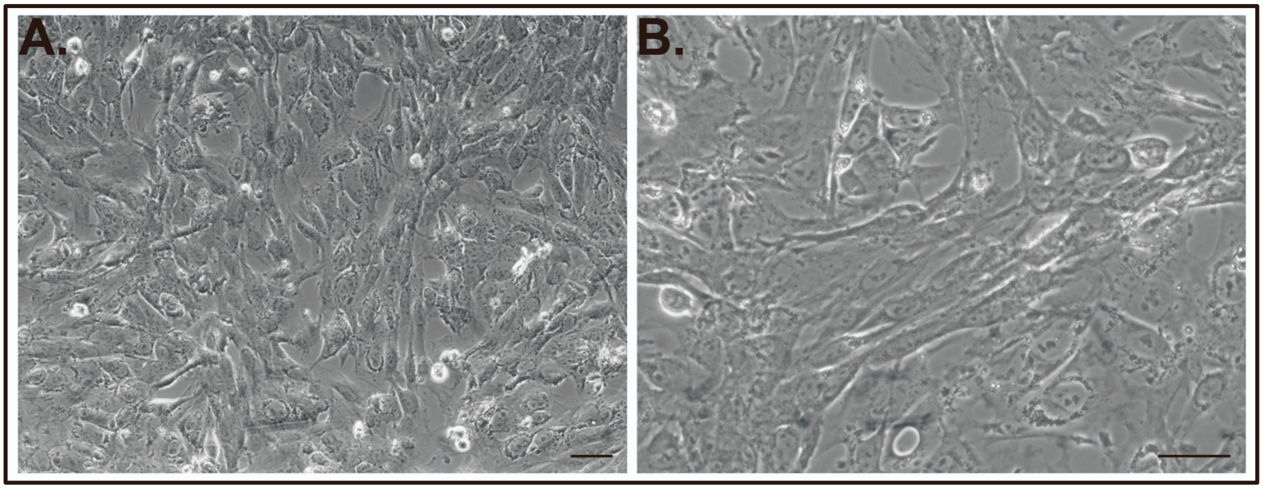

Supplementary Figure 4. Phase-contrast microscopy of human embryonic stem cell-derived endothelial mesenchymal transformed corneal endothelial cells

The images of cells at passage 4 are captured at (A) 10x, and (B) 20x magnification. Scale bars, 50 μm.

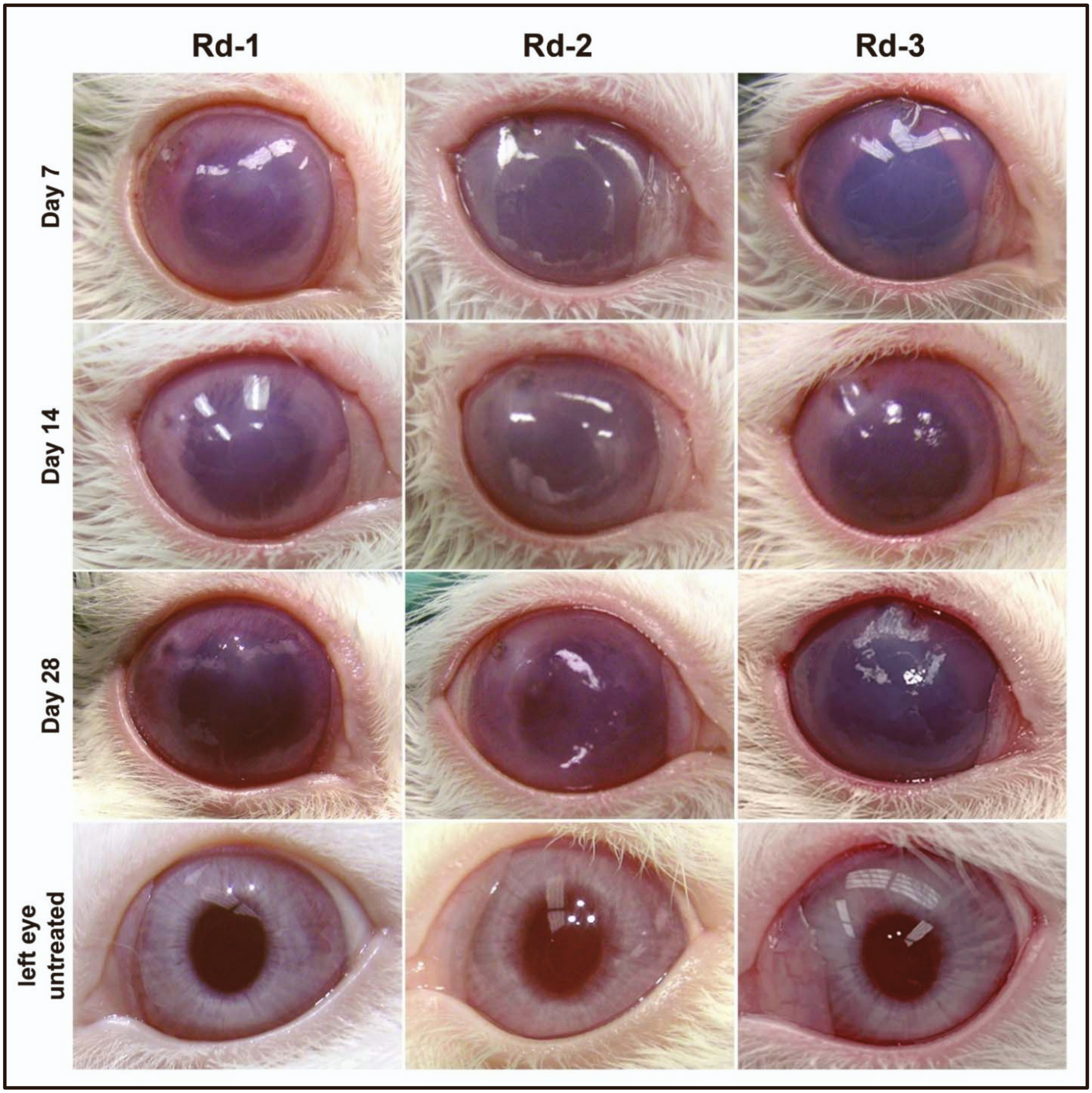

Supplementary Figure 5. Illustration of lack of corneal transparency after injection of cryopreserved human embryonic stem cell-derived endothelial mesenchymal transformed corneal endothelial cells (hESC-derived EnMT-CECs) in rabbits

Representative images of the cryopreserved hESC-derived EnMT-CEC injected right eyes on days 7, 14, and 28, and untreated left eyes.

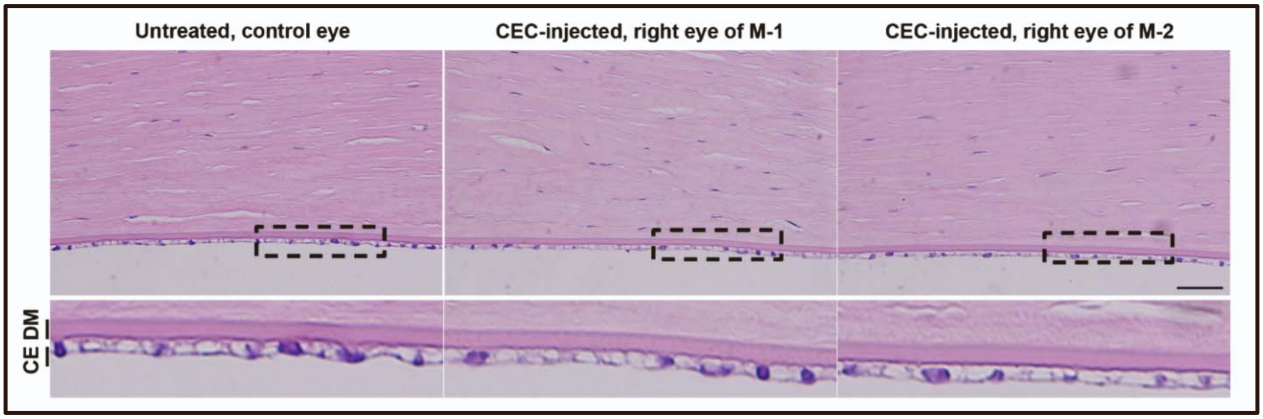

Supplementary Figure 6. Hematoxylin and eosin (H&E) staining of cryopreserved human embryonic stem cell (hESC)-derived corneal endothelial cell (CEC) injected monkey corneas

Monkey corneas of untreated control eye and cryopreserved hESC-derived CEC injected right eyes of M-1 and M-2. The monkeys (M-1, and M-2) represent the injection model where hESC-derived CECs were injected immediately after the removal of the central corneal endothelium. The boxed areas are enlarged and shown in panels below. The images are captured at 20x magnification. Scale bar, 50 µm.

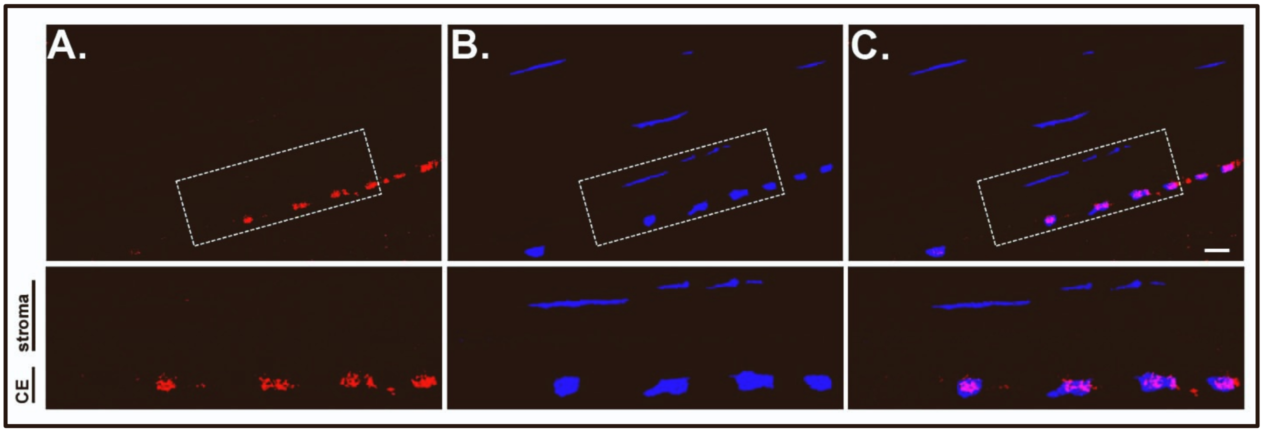

Supplementary Figure 7. Immunohistochemical analysis of the human antigen in cryopreserved human embryonic stem cell (hESC)-derived corneal endothelial cell (CEC) injected monkey corneas

The analysis confirms that the regenerated corneal endothelium (CE) of the right eye of the monkey (M-1) consists of cryopreserved hESC-derived CECs. (A) Immunostaining of human-specific nucleoli antibody. (B) Cell nuclei are counterstained with DAPI (4′,6-diamidine-2′-phenylindole dihydrochloride). (C) Merge of images shown in a and b. The corneal stroma consists of resident keratocytes, which serve as an internal negative control, and importantly, do not show reactivity with the human-specific antibody. M-1 represents the injection model monkey where hESC-derived CECs were injected immediately after the removal of the central CE. The boxed areas are enlarged and shown in panels below. The images are captured at 40x magnification. Scale bar, 10 μm.

Supplementary Table 1: The summary of the necropsy examination of rabbits injected with cryopreserved human embryonic stem cell (hESC)-derived corneal endothelial cells (CECs)

Anatomic diagnosis	Necropsy examination of injection model rabbits 9 months postinjection			Necropsy examination of injury-injection model rabbits 18 months postinjection	
	**R-1**	**R-2**	**R-3**	**R-4**	**R-5**
**Adrenal glands**	Unremarkable	Unremarkable	Unremarkable	Unremarkable	Unremarkable
**Bone marrow**	Unremarkable	Unremarkable	Unremarkable	Unremarkable	Unremarkable
**Brain**	Unremarkable	Unremarkable	Unremarkable	Unremarkable	Unremarkable
**Gall bladder**	Unremarkable	Unremarkable	Unremarkable	Unremarkable	Unremarkable
**Heart**	Unremarkable	Unremarkable	Unremarkable	Unremarkable	Unremarkable
**Kidneys**	Unremarkable similar in size and shape	Unremarkable similar in size and shape	Unremarkable similar in size and shape	Unremarkable, similar in size and shape	Unremarkable, similar in size and shape
**Liver**	Unremarkable	Unremarkable	Unremarkable	Unremarkable	Unremarkable
**Lungs**	Unremarkable	Unremarkable	Unremarkable	Unremarkable	Unremarkable
**Optic nerves**	Unremarkable	Unremarkable	Unremarkable	Unremarkable	Unremarkable
**Pituitary**	Unremarkable	Unremarkable	Unremarkable	Unremarkable	Unremarkable
**Spleen**	Unremarkable	Unremarkable	Unremarkable	Unremarkable	Unremarkable
**Urinary bladder**	Unremarkable	Unremarkable	Unremarkable	Unremarkable	Unremarkable

Note: The three rabbits (R-1, R-2, and R-3) represent the injection model (hESC-derived CECs injected immediately after the removal of the central corneal endothelium (CE)), and the two rabbits (R-4, and R-5) represent the injury-injection model (hESC-derived CECs injected four days after the removal of the central CE).

Supplementary Table 2: The summary of the necropsy examination of monkeys injected with cryopreserved human embryonic stem cell (hESC)-derived corneal endothelial cells (CECs)

Anatomic diagnosis	Necropsy examination of injection model monkeys ≥12 months postinjection			Necropsy examination of injury-injection model monkeys 21 months postinjection	
	**M-1**	**M-2**	**M-3**	**M-4**	**M-5****
**Adrenal glands**	Unremarkable	Unremarkable	Unremarkable	Unremarkable	Unremarkable
**Bone marrow**	Unremarkable	Unremarkable	Unremarkable	Unremarkable	Unremarkable
**Brain**	Unremarkable	Unremarkable	Unremarkable	Unremarkable	Unremarkable
**Gall bladder**	Unremarkable	Unremarkable	Unremarkable	Unremarkable	Unremarkable
**Heart**	Unremarkable	Unremarkable	Unremarkable	Unremarkable	Unremarkable
**Kidneys**	Unremarkable, similar in size and shape	Unremarkable, similar in size and shape	Unremarkable, similar in size and shape	Unremarkable, similar in size and shape	Unremarkable, similar in size and shape
**Liver**	Unremarkable	Unremarkable	Unremarkable	Unremarkable	Unremarkable
**Lungs**	Unremarkable	Unremarkable	Unremarkable	Unremarkable	Unremarkable
**Optic nerves**	Unremarkable, smooth, and symmetric	Unremarkable, smooth, and symmetric	Unremarkable, smooth, and symmetric	Unremarkable, smooth, and symmetric	Unremarkable, smooth, and symmetric
**Pituitary**	Unremarkable	Unremarkable	Unremarkable	Unremarkable	Unremarkable
**Spleen**	Unremarkable	Unremarkable	Unremarkable	Unremarkable	Unremarkable
**Urinary bladder**	Unremarkable	Unremarkable	Unremarkable	Unremarkable	Unremarkable

Note: The monkeys, M-1, M-2, and M-3 represent the injection model (hESC-derived CECs injected immediately after the removal of the central corneal endothelium (CE)), and the monkeys M-4, and M-5 represent the injury-injection model (hESC-derived CECs injected two days after the removal of the central CE). The injection model monkeys (M1 & M2) were housed for 12 months except for M3 that was housed for 26 months postinjection before euthanization and subsequent necropsy examination. **M-5 exhibited the clinical signs of bloody mucoid diarrhea at the euthanization time point and fecal PCR was positive for Shigella and Campylobacter. Importantly, other significant findings were not identified. An examination before euthanization confirmed that the clinical characteristics of the M-3, M-4, and M-5 were in line with the results presented in figures 5, and 6 i.e., the cornea of the injected (right) eye is transparent, and the central corneal thickness of the injected (right) eye remained comparable (± 20 μm) to the untreated (left) eye.

Supplementary Data 1: A complete list of genes identified in non-cryopreserved human embryonic stem cell-derived corneal endothelial cells

The gene expression data were normalized by calculating the FPKM (Fragments per kilobase per million mapped reads) for each gene. A cut-off value of ≥ 1 FPKM was established as an expression threshold for each gene.

Supplementary Data 2: A complete list of genes identified in cryopreserved human embryonic stem cell-derived corneal endothelial cells

The gene expression data were normalized by calculating the FPKM (Fragments per kilobase per million mapped reads) for each gene. A cut-off value of ≥ 1 FPKM was established as an expression threshold for each gene.

Experimental Procedures

Characterization of H9 human embryonic stem cell (hESC)-derived corneal endothelial cells (CECs) by phase-contrast microscopy and quantitative real-time PCR (qRT-PCR)

Phase-contrast microscopy was performed using a Zeiss inverted microscope (Zeiss, Germany), equipped with Q-Capture imaging software (QImaging, Surrey, BC, Canada). Total RNA for qRT-PCR from H9 hESCs, hESC-derived CECs at day 20, and cryopreserved hESC-derived CECs was extracted using TRIzol reagent (Invitrogen; Carlsbad, CA). First-strand cDNA synthesis was completed using the Superscript III kit (Invitrogen) according to the manufacturer's instructions.

The expression of corneal endothelium (CE)-associated markers (*AQP1, ATP1A1, TJP1, COL4A1, COL4A3, COL8A1, COL8A2, FOXC1, and SLC16A3*) were quantitated using qRT-PCR as described (Ali et al., 2018). The primers were designed using a real-time PCR tool (Integrated DNA Technologies; Coralville, IA) and are available upon request. qRT-PCR was performed on STEP ONE ABI Real-Time PCR System (Applied Biosystems; Foster City, CA). Delta-delta C_T method was used to determine the relative expression, normalized against *GAPDH* expression (Livak and Schmittgen, 2001).

Characterization of hESC-derived CECs by immunocytochemistry

The hESC-derived CECs were examined by immunocytochemistry. Briefly, the cells were fixed with 4% paraformaldehyde (PFA) for 15 minutes followed by blocking with 5% bovine serum albumin (MilliporeSigma; Burlington, MA). The cells were first incubated with 1:100 zona occludens-1 (ZO-1; catalog # 13663s; Cell Signaling Technology; Danvers, MA) primary antibody overnight at 4°C. The cells were next treated with 1:100 FITC-conjugated goat anti-rabbit IgG (catalog # AP307F; MilliporeSigma) secondary antibody for 2 hours at room temperature. The nuclei were counterstained with DAPI (4′,6-diamidine-2′-phenylindole dihydrochloride; MilliporeSigma). The images of mounted cells were captured using an Olympus

LX81 microscope (Olympus, Tokyo, Japan) equipped with software (Slidebook Software 3i; Denver, CO) and prepared using image-editing software (Adobe Photoshop CS5; Adobe Systems, Inc., San Jose, CA).

Next-generation RNA sequencing (RNA-Seq) of hESC-derived CECs

Next-generation RNA-Seq of hESC-derived CECs at day 20 (non-cryopreserved) and cryopreserved hESC-derived CECs (cryopreserved for 40 days in liquid nitrogen) was performed. Briefly, three biological replicates for each of non-cryopreserved hESC-derived CECs and cryopreserved hESC-derived CECs were used for RNA-Seq library preparation. Total RNA was isolated from each sample using TRIzol reagent (Invitrogen). The extracted RNA was examined using a NanoDrop Lite spectrophotometer (Thermo Fisher Scientific) and RNA 6000 Pico kit on an Agilent 2100 Bioanalyzer (Agilent; Palo Alto, CA). RNA-Seq library preparation, next-generation sequencing, and analysis of the RNA-Seq datasets including reads mapping to reference genome, quantification of gene expression, differential gene expression analysis, and correlations were performed commercially by Novogene Corporation Inc (Sacramento, CA). All raw sequencing data reported in this manuscript have been deposited in NCBI's Gene Expression Omnibus (Edgar et al., 2002).

References

Ali, M., Khan, S.Y., Vasanth, S., Ahmed, M.R., Chen, R., Na, C.H., Thomson, J.J., Qiu, C., Gottsch, J.D., and Riazuddin, S.A. (2018). Generation and Proteome Profiling of PBMC-Originated, iPSC-Derived Corneal Endothelial Cells. Invest Ophthalmol. Vis. Sci. 59, 2437-2444.

Edgar, R., Domrachev, M., and Lash, A.E. (2002). Gene Expression Omnibus: NCBI gene expression and hybridization array data repository. Nucleic Acids Res. 30, 207-210.

Livak, K.J., and Schmittgen, T.D. (2001). Analysis of relative gene expression data using real-time quantitative PCR and the 2(-Delta Delta C(T)) Method. Methods 25, 402-408.

Mortazavi, A., Williams, B.A., McCue, K., Schaeffer, L., and Wold, B. (2008). Mapping and quantifying mammalian transcriptomes by RNA-Seq. Nat. Methods 5, 621-628.
