# Supplementary material for: Pluripotent stem cell–derived corneal endothelial cells as an alternative to donor corneal endothelium in keratoplasty
Source: Stem Cell Reports. 2021 Aug 5;16(9):2320–35. doi: 10.1016/j.stemcr.2021.07.008 (PMC8452521; doi:10.1016/j.stemcr.2021.07.008)
Supplement: Document S2. Article plus supplemental information [file mmc4.pdf]

Stem Cell Reports
Article
OPEN ACCESS

Pluripotent stem cell–derived corneal endothelial cells as an alternative to donor corneal endothelium in keratoplasty

Muhammad Ali,[1] Shahid Y. Khan,[1] John D. Gottsch,[1] Eric K. Hutchinson,[2] Aisha Khan,[3] and S. Amer Riazuddin[1,*]
[1]The Wilmer Eye Institute, Johns Hopkins University School of Medicine, Baltimore, MD 21287, USA
[2]Department of Molecular and Comparative Pathobiology, Johns Hopkins University School of Medicine, Baltimore, MD 21205, USA
[3]Interdisciplinary Stem Cell Institute, University of Miami Miller School of Medicine, Miami, FL 33136, USA
*Correspondence: riazuddin@jhmi.edu
https://doi.org/10.1016/j.stemcr.2021.07.008

SUMMARY

Here, we evaluate the efficacy of cryopreserved human embryonic stem cell (hESC)-derived corneal endothelial cells (CECs) to form a functional monolayer of corneal endothelium (CE) in rabbits and monkeys. We injected cryopreserved hESC-derived CECs into the anterior chamber of rabbits and monkeys either immediately after mechanical scraping of the central CE or a few days later when corneal edema developed. All preclinical models developed deturgesced and clear corneas following the injection of cryopreserved hESC-derived CECs and remained comparable to the corneas of the untreated eye. Confocal scanning microscopy confirmed an intact structure of hexagonal/polygonal cells and immunohistochemical analysis illustrated a monolayer expressing barrier and pump function proteins in the regenerated CE. The necropsy examination confirmed no remarkable change in multiple tissues assessed for teratoma formation. In conclusion, our data demonstrate the efficacy of cryopreserved hESC-derived CECs to form a functional CE on the denuded Descemet's membrane.

INTRODUCTION

The corneal endothelium (CE) is the innermost monolayer of the cornea and is composed of hexagonal/polygonal cells that maintain corneal transparency by mediating hydration through the barrier and pump functions (Bonanno, 2012). An adult CE is reported to have a corneal endothelial cell (CEC) density of approximately 2,500 cells per square millimeter (cells/mm^2) and the physiological functioning of the CE may be substantially compromised below a CEC density of 500 cells/mm^2, which can lead to corneal edema and vision loss (Tan et al., 2012).

Currently, the only treatment modality to restore corneal endothelial dysfunction mediated vision loss is human donor tissue–dependent surgical transplantation, whereas full and partial-thickness keratoplasty techniques have been effective in restoring vision in patients affected with corneal endothelial dystrophies. However, in many areas of the world, transplantable-grade donor CE is limited. A global survey of corneal transplant surgery reported a considerable shortage of corneal transplant tissue, with only one cornea available for every 70 patients in need (Gain et al., 2016).

CEC injection has received recent attention to address the worldwide shortage of transplantable-grade donor CE. A few preclinical studies have been reported in rabbit and monkey models to assess the technical efficacy of CEC-injection therapy (Okumura et al., 2016; Shen et al., 2017). Kinoshita and colleagues have reported the successful treatment of 11 patients suffering from bullous keratopathy and Fuchs endothelial corneal dystrophy by injecting cultured human CECs in combination with a rho-associated protein kinase (ROCK) inhibitor into the anterior chamber (Kinoshita et al., 2018).

Pluripotent stem cells have the potential to differentiate into several cell types including CECs. A number of investigators have reported the generation of CECs from human embryonic stem cells (hESCs) and induced pluripotent stem cells (iPSCs) (McCabe et al., 2015; Song et al., 2016; Wagoner et al., 2018; Zhao and Afshari, 2016). We previously reported the differentiation of peripheral blood mononuclear cell (PBMC)-originated, iPSCs to CECs that share a similar proteome profile with human CE (Ali et al., 2018a). We further confirmed that hESC- and iPSC-derived CECs have largely equivalent transcriptome profiles (Ali et al., 2018b).

Here, we examine the efficacy of xeno-free cryopreserved hESC-derived CECs as an alternative to donor corneal transplantation in animal models. We demonstrate that cryopreservation does not affect the viability and transcriptome profile of hESC-derived CECs. Cryopreserved hESC-derived CECs form a functional CE when injected into the anterior chamber of both rabbit and monkey corneal endothelial dysfunction models. A complete necropsy examination confirmed no abnormal pathology including teratomas in multiple tissues. To the best of our knowledge, this is the first report examining the efficacy of cryopreserved hESC-derived CECs as an alternative to donor corneal tissue to restore functional corneal integrity and clarity in animal models.

This is an open access article under the CC BY license (http://creativecommons.org/licenses/by/4.0/).

RESULTS

Characterization of non-cryopreserved and cryopreserved hESC-derived CECs

We previously reported proteome profiling of PBMC-originated, iPSC-derived CECs, and comparative transcriptome analysis of hESC- and iPSC-derived CECs (Ali et al., 2018a, 2018b). We employed the same 20-day procedure to generate hESC-derived CECs under feeder- and xeno-free conditions. The morphological examination of hESC-derived CECs at day 20 exhibited a hexagonal/polygonal morphology (Figure S1). The examination of hESC-derived CECs for expression of zona-occludens-1 (ZO-1) by immunostaining illustrated expression of ZO-1 at cell boundaries (Figures 1A–1C) and confirmed the tightly packed layer of hESC-derived CECs generated under xeno-free conditions.

To examine the possible effects of cryopreservation on hESC-derived CECs, we examined the expression profile of hESC-derived CEC pelleted cells stored at $-80^{\circ}C$ (termed non-cryopreserved hESC-derived CECs) and hESC-derived CECs cryostored in three distinct pools in liquid nitrogen for 40 days (termed cryopreserved hESC-derived CECs). We first performed quantitative real-time PCR (qRT-PCR) for CE-associated markers (*AQP1, ATP1A1, TJP1, COL4A1, COL4A3, COL8A1, COL8A2, FOXC1,* and *SLC16A3*) that suggested indistinguishable expression pattern in non-cryopreserved and cryopreserved hESC-derived CECs (Figure 1D).

We next performed a comparative analysis employing next-generation RNA sequencing (RNA-Seq) of the non-cryopreserved and the cryopreserved hESC-derived CECs. These analyses identified the expression ($\geq$1 FPKM; fragments per kilobase per million mapped reads) of 14,445 and 14,022 genes in the non-cryopreserved and the cryopreserved hESC-derived CECs, respectively (Data S1 and S2) with a high correlation (Pearson coefficient = 0.975) (Figure 1E). Comparative analysis of the non-cryopreserved and cryopreserved hESC-derived CEC transcriptomes revealed 13,423 (>96%) genes shared in both transcriptomes (Figure 1F), which supports the notion that cryopreservation, at least up to 40 days in liquid nitrogen, does not affect the expression profile of hESC-derived CECs.

Injection of cryopreserved hESC-derived CECs

To examine the efficacy of cryopreserved hESC-derived CECs to form a functional CE in New Zealand white rabbits (*Oryctolagus cuniculus*) and rhesus monkeys (*Macaca mulatta*), we investigated four different preclinical models. These included (1) injection model in rabbits, (2) injury-injection model in rabbits, (3) injection model in monkeys, and (4) injury-injection model in monkeys. The two models in rabbits were developed by mechanical scraping of CE followed by injection of hESC-derived CECs either immediately after scraping of the CE (injection model in rabbits) or 4 days later when corneal edema had developed (injury-injection model in rabbits). Similar models in non-human primates were developed by mechanical scraping of CE in monkeys followed by injection of hESC-derived CECs either immediately after scraping of the CE (injection model in monkeys) or 2 days later when corneal edema had developed (injury-injection model in monkeys).

Injection model in rabbits

The model was developed by the mechanical scraping of CE from the Descemet's membrane (DM) using a silicon needle (please see experimental procedures for details). To investigate the extent of damage induced by mechanical scraping and to confirm that the scraped area is devoid of CECs, Calcein-AM staining was performed in control rabbits prior to the injection model. Staining confirmed that the scraped area was devoid of CECs after mechanical scraping of the CE (Figures S2A–S2D). We further examined the removal of CE and damage, if any, to the DM by hematoxylin and eosin (H&E) staining (in controls prior to the injection model in rabbits). The H&E staining confirmed that the scraped area was devoid of CECs with an intact DM following mechanical scraping of CE (Figures S2E–S2H).

Once we confirmed that mechanical scraping with the silicon needle removes CE and does not induce damage to the DM, we injected a total of 750,000 cryopreserved hESC-derived CECs supplemented with 100 μM of ROCK inhibitor in the anterior chamber of the right eye of three rabbits (R-1, R-2, and R-3) after mechanical scraping of the central CE while the left untreated eye served as a control. We monitored both eyes in all three rabbits for corneal thickness and transparency at constant intervals. In the initial days postinjection, the corneal thickness of the injected (right) eye increased 2–3 times compared with the untreated (left) eye accompanied by loss of corneal transparency in the injected eye. However, the edema resolved, and corneas attained transparency 14–21 days postinjection in all three rabbits (Figure 2).

Confocal (confoscan4 scanning; Nidek Technologies, Albignasego PD, Italy) images show an intact layer of hexagonal/polygonal cells in the cryopreserved hESC-derived CEC-injected rabbit corneas (Figure 3A). The CEC density of the injected (right) eye was >80% of the CEC density of the untreated (left) eye and remained stable (±2%) at the 6- and 9-month postinjection time points (Figure 3B). Pachymetry measurements confirmed that the central corneal thickness of the injected (right) eye remained comparable (±20 μm) to the untreated (left) eye at the 6- and 9-month postinjection time points (Figure 3C). Intraocular pressure (IOP) of the injected (right) and untreated (left) eyes remained within 9–15 mm Hg in all three rabbits.

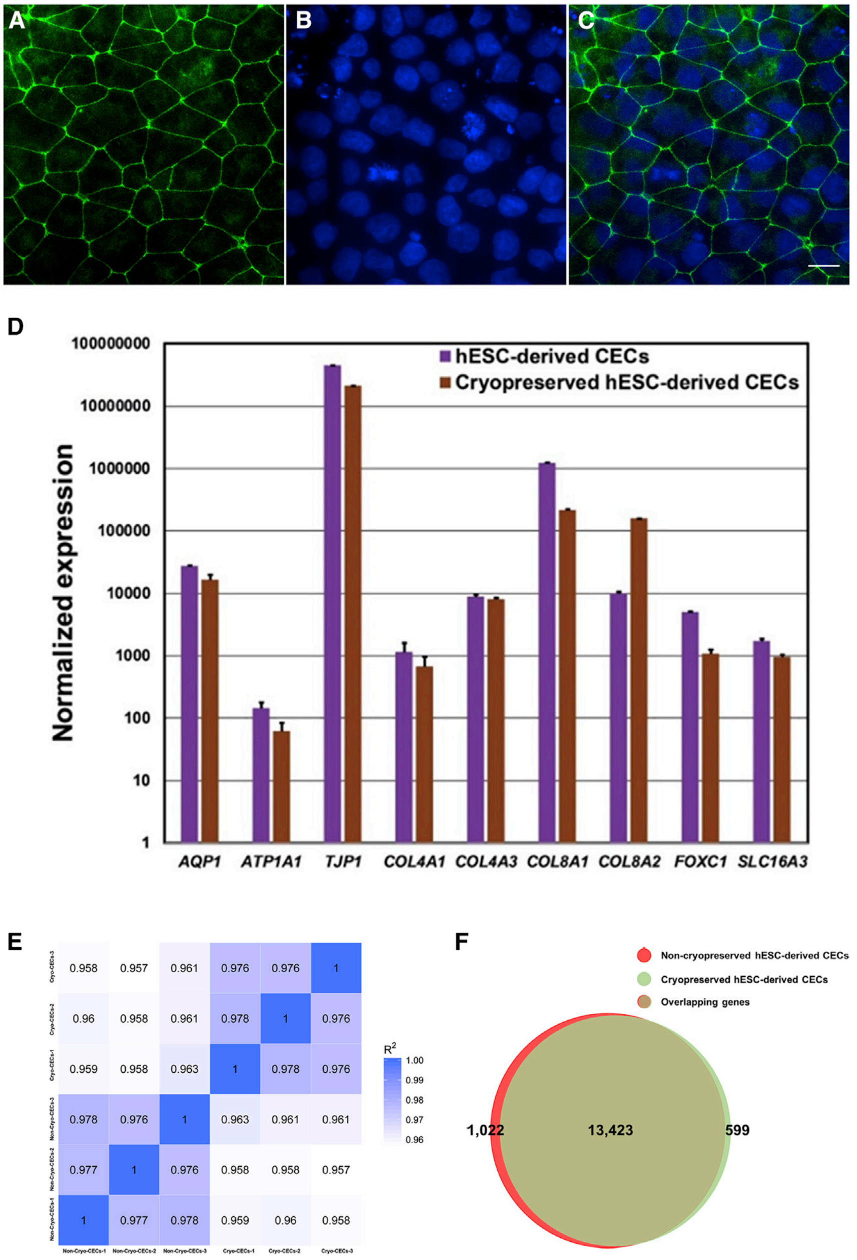

Figure 1. Characterization of cryopreserved human embryonic stem cell (hESC)-derived corneal endothelial cells (CECs)

(A–C) Immunostaining for zona occludens-1 (ZO-1; a tight-junction protein). Cell nuclei were counterstained with DAPI (4′,6-diamidine-2′-phenylindole dihydrochloride). Images were captured at ×60 magnification. Scale bar, 10 μm.

(D) Quantitative real-time PCR of corneal endothelium (CE)-associated markers (*AQP1, ATP1A1, TJP1, COL4A1, COL4A3, COL8A1, COL8A2, FOXC1,* and *SLC16A3*) in cryopreserved and non-cryopreserved hESC-derived CECs.

(E and F) Next-generation RNA sequencing of the non-cryopreserved and the cryopreserved hESC-derived CECs revealed a high correlation (Pearson coefficient = 0.975) among the datasets with 13,423 (>96%) genes shared in both transcriptomes.

Note: Error bars represent standard deviation in each data set examined in four independent replicates.

The H&E staining of cryopreserved hESC-derived CEC-injected rabbit corneas revealed an intact monolayer of tightly packed CECs that were adherent to the DM (Figure S3). The immunohistochemical (IHC) analysis of ZO-1, ATPase sodium/potassium subunit alpha1 (Na^+/K^+ ATPase α1), and N-cadherin illustrated a monolayer of cells expressing barrier and pump function proteins in the regenerated CE (Figure 4).

It has been documented that rabbit CE has a proliferation ability as opposed to human CE (Van Horn et al., 1977). Although multiple studies have shown intracameral injection of cultured CECs into the anterior chamber of bullous keratopathy rabbit models result in regeneration of the CE (Peh et al., 2019; Shen et al., 2017), we sought evidence to confirm that a functional CE is only possible with the injection of CECs and not other cell types, including derivatives of CECs. We, therefore, prepared hESC-derived endothelial-mesenchymal transformed CECs (EnMT-CECs) by a procedure described by Yamashita et al. (2018). The morphology of the hESC-derived EnMT-CECs was confirmed by phase-contrast microscopy (Figures S4A and S4B). We cryopreserved the hESC-derived EnMT-CECs similar to the cryopreservation procedure adopted for the hESC-derived CECs and

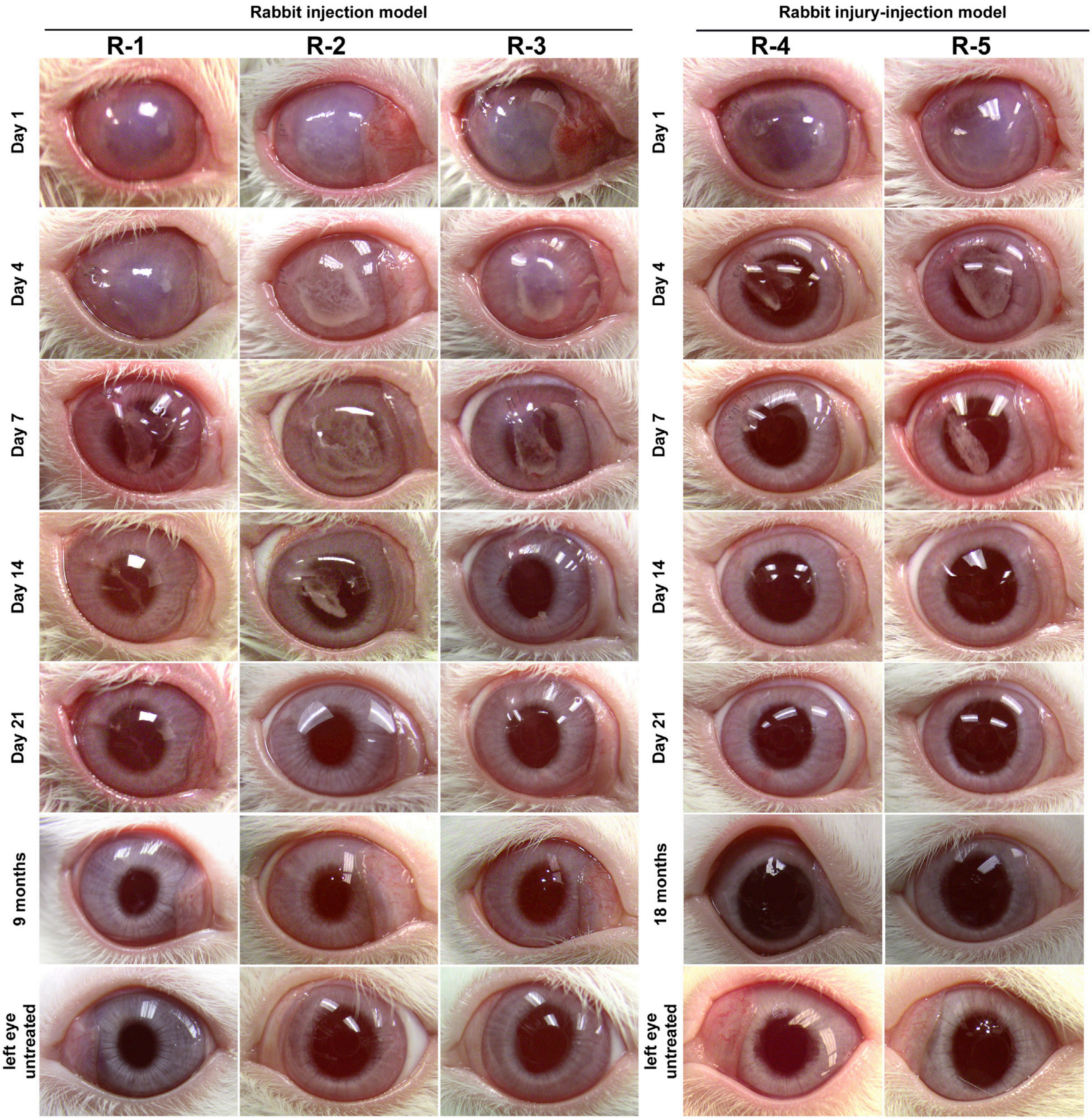

Figure 2. Illustration of corneal transparency after the injection of cryopreserved human embryonic stem cell (hESC)-derived corneal endothelial cells (CECs) in rabbits

Representative images of cryopreserved hESC-derived CEC injected right eye on days 1, 4, 7, 14, and 21, and 9 months (R-1, R-2, and R-3) or 18 months (R-4 and R-5) and untreated left eyes. The three rabbits (R-1, R-2, and R-3) represent the injection model (hESC-derived CECs injected immediately after the removal of central corneal endothelium [CE]), and the two rabbits (R-4 and R-5) represent the injury-injection model (hESC-derived CECs injected 4 days after the removal of the central CE).

injected cryopreserved hESC-derived EnMT-CECs with 100 μM ROCK inhibitor in three rabbits (Rd-1, Rd-2, and Rd-3). The injected hESC-derived EnMT-CECs were unable to reverse the induced corneal edema 28 days postinjection (Figure S5). This is in sharp contrast to the corneas of cryopreserved hESC-derived CEC-injected eyes that attained transparency within three weeks postinjection (Figure 2). It is noteworthy that hESC-derived EnMT-CECs are

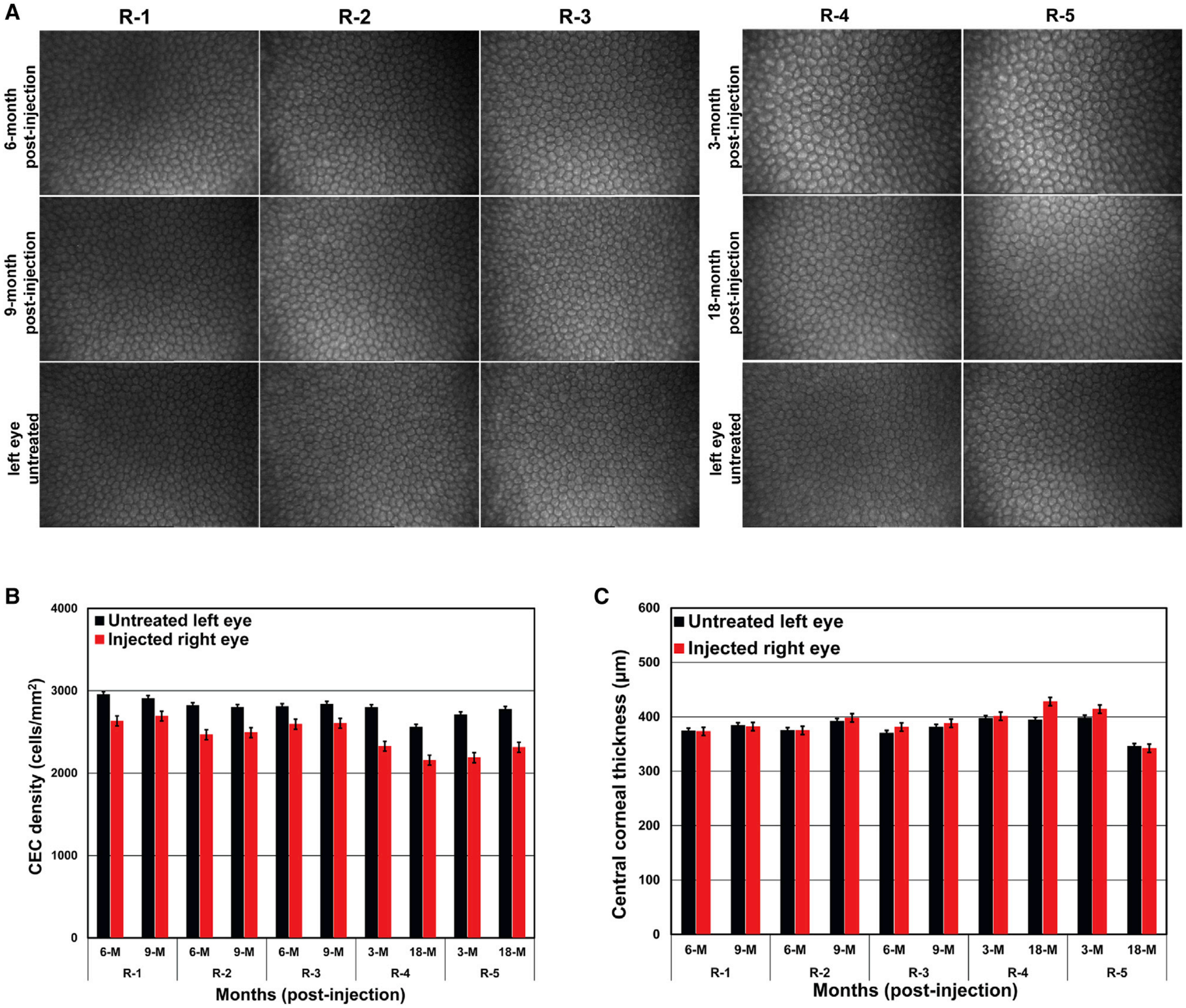

Figure 3. Evaluation of the regenerated corneal endothelium (CE) after injection of cryopreserved human embryonic stem cell (hESC)-derived corneal endothelial cells (CECs) in rabbits

The three rabbits (R-1, R-2, and R-3) represent the injection model (hESC-derived CECs injected immediately after the removal of the central CE), and the two rabbits (R-4 and R-5) represent the injury-injection model (hESC-derived CECs injected 4 days after the removal of the central CE).

(A) The CE of hESC-derived CEC-injected right eyes of R-1, R-2, and R-3 at 6 and 9 months postinjection and of R-4 and R-5 at 3 and 18 months postinjection along with untreated left eyes were examined using a confoscan4 scanning microscope.

(B) Illustration of the CEC density (cells/mm^2) of hESC-derived CEC-injected right eyes and untreated left eyes of R-1, R-2, and R-3 at 6 and 9 months postinjection, and of R-4 and R-5 at 3 and 18 months postinjection.

(C) Demonstration of the central corneal thickness of hESC-derived CEC-injected right eyes and untreated left eyes of R-1, R-2, and R-3 at 6 and 9 months postinjection, and of R-4 and R-5 at 3 and 18 months postinjection.

Note: Error bars represent standard deviation in each data set examined in four independent replicates.

generated from the hESC-derived CECs (please see the methods). Taken together, these data confirm that only hESC-derived CECs and not even derivatives of CECs injected into the anterior chamber can form a functional CE in the rabbit injection models.

Injury-injection model in rabbits

Next, we determined if the injection of cryopreserved hESC-derived CECs can form a functional monolayer of CE on an edematous cornea. We removed the CE as described above and housed the rabbits for 4 days while

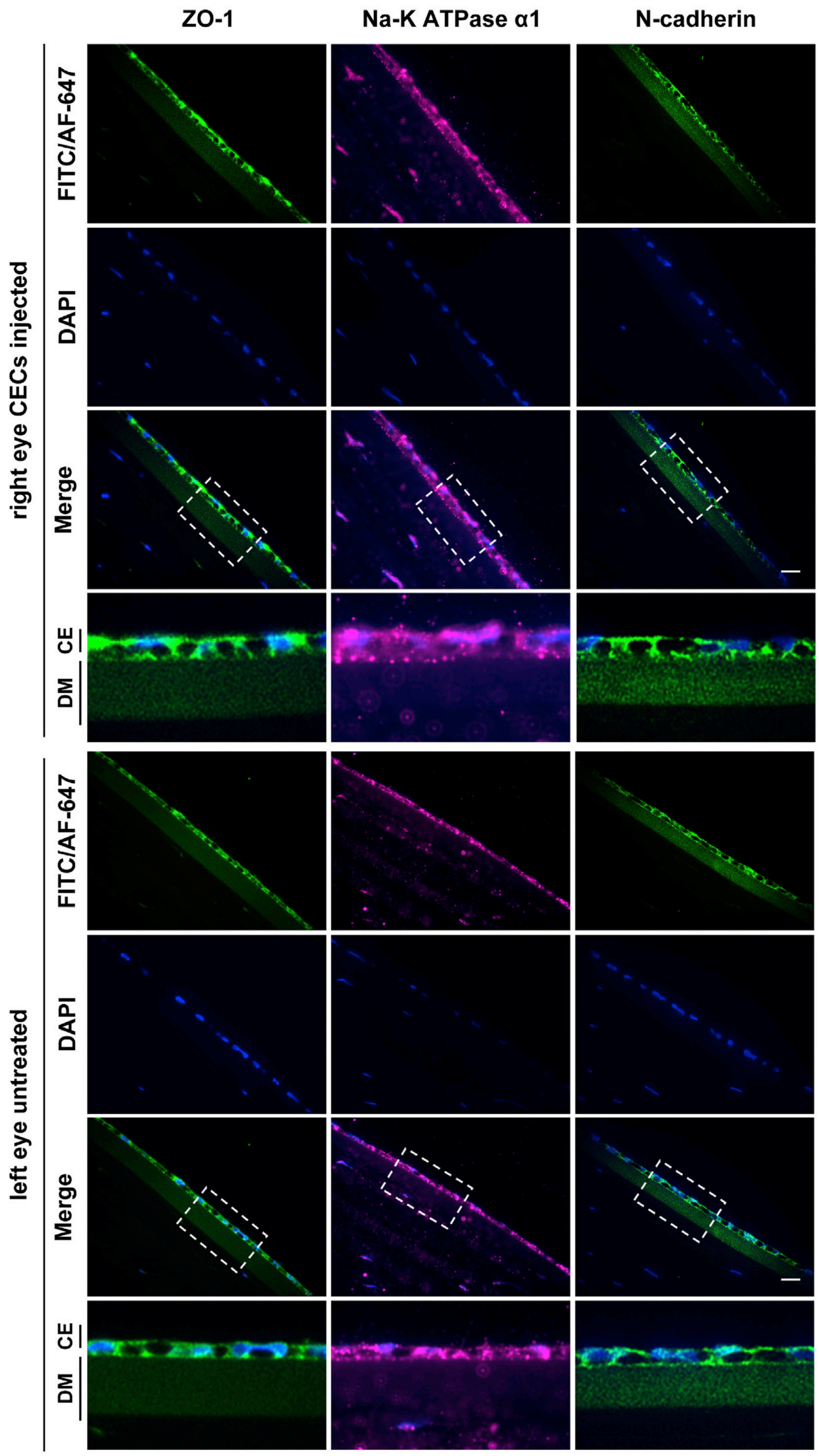

Figure 4. Immunohistochemical analysis of tight junction, pump function, and structural-integrity related proteins in the regenerated corneal endothelium (CE) in rabbits

The analysis of cryopreserved human embryonic stem cell (hESC)-derived corneal endothelial cell (CEC)-injected right eye and untreated left eye of rabbit, R-2 representing the injection model (hESC-derived CECs injected immediately after the removal of central corneal endothelium) after euthanization 9 months post-injection. Immunostaining of zona-occludens-1 (ZO-1), ATPase sodium/potassium subunit alpha1 (Na^+/K^+ ATPase α1), and N-cadherin was performed as described in experimental procedures. Cell nuclei were counterstained with DAPI (4′,6-diamidine-2′-phenylindole dihydrochloride). The boxed areas are enlarged and shown in the panels below. Note: The images were captured at ×60 magnification. Scale bar, 10 μm.

applying ointment (tobramycin and dexamethasone) three times a day to reduce infection and inflammation (according to protocols approved by the Animal Care and Use Committee [ACUC] of Johns Hopkins University School of Medicine). On day 4 post CE removal, we injected 750,000 cryopreserved hESC-derived CECs supplemented with 100 μM of ROCK inhibitor in the anterior chamber of the right eye of two rabbits (R-4 and R-5), i.e., injury-injection model in rabbits, and monitored the recovery and formation of injected CEC-derived functional CE. Similar to rabbits in the injection model, the corneal thickness of the injected (right) eye increased 2–3 times compared with the untreated (left) eye accompanied by loss of corneal transparency. However, the edema resolved, and corneas attained transparency 14–21 days postinjection in both rabbits (Figure 2).

Confocal images demonstrate an intact layer of hexagonal/polygonal cells in the cryopreserved hESC-derived CEC-injected rabbit corneas (Figure 3A). Similar to rabbits in the injection model, the CEC density of the injected (right) eye was >80% of the CEC density of the untreated (left) eye and remained stable (±2%) at the 3- and 18-month postinjection time points (Figure 3B). Likewise, pachymetry measurements confirmed that the central corneal thickness of the injected (right) eye remained comparable (±20 μm) to the untreated (left) eye at the 3- and 18-month postinjection time points (Figure 3C). IOP of the injected (right) and untreated (left) eyes remained within 9–15 mm Hg in both rabbits.

Injection model in monkeys

We next examined the efficacy of cryopreserved hESC-derived CECs to form a functional CE in non-human primates. We injected a total of 1×10^6 cryopreserved hESC-derived CECs supplemented with 100 μM of ROCK inhibitor in the anterior chamber of the right eyes of three monkeys (M-1, M-2, and M-3) after mechanical scraping of the central CE while the untreated (left) eyes served as a control. Similar to the rabbit models, after the injection of the cryopreserved hESC-derived CECs, the corneal thickness of the injected (right) eye increased 2 to 3 times compared with the untreated (left) eye accompanied by loss of corneal transparency in the injected eye (Figure 5). The edema resolved and corneas attained transparency 7–8 days postinjection in all three monkeys (Figure 5).

Confocal images show an intact layer of hexagonal/polygonal cells in the cryopreserved hESC-derived CEC-injected monkey corneas (Figure 6A). The CEC density of the injected (right) eye was >80% of the CEC density of the untreated (left) eye and remained stable (±2%) at the 6- and 12-month postinjection time points (Figure 6B). The pachymetry measurements confirmed that the central corneal thickness of the injected eye remained comparable (±20 μm) to the untreated (left) eye at the 6- and 12-month postinjection time points (Figure 6C). IOP of the injected (right) and untreated (left) eyes remained within 9–14 mm Hg in all three monkeys.

The H&E staining of cryopreserved hESC-derived CEC-injected monkey corneas demonstrated an intact monolayer of tightly packed CECs adherent to DM (Figure S6). Moreover, H&E staining showed that the thickness of hESC-derived CEC-injected corneas was similar to corneas in control (left) eyes (Figure S6). The IHC analysis of ZO-1, Na^+/K^+ ATPase α1, and N-cadherin illustrated a monolayer of cells expressing barrier and pump function proteins in the regenerated CE (Figure 7).

Injury-injection model in monkeys

As above in rabbits, we asked if the injection of cryopreserved hESC-derived CECs can form a functional monolayer of CE on a swollen cornea mimicking corneal edema in monkeys. We removed CE as described above and housed the monkeys with denuded DM for 2 days while applying ointment (tobramycin and dexamethasone) every 24 h to prevent infection and inflammation according to the protocol approved by the ACUC of Johns Hopkins University School of Medicine. On day 2 after the removal of the central CE, we injected 1×10^6 cryopreserved hESC-derived CECs supplemented with 100 μM of ROCK inhibitor in the anterior chamber of the right eyes of two monkeys (M-4 and M-5), and monitored the recovery and formation of a CEC-derived functional CE. Similar to monkeys in the injection model, the corneal thickness of the injected (right) eye increased 2 to 3 times compared with the untreated (left) eye accompanied by loss of corneal transparency. However, the edema resolved, and corneas attained transparency 7-8 days postinjection in both monkeys (Figure 5).

Confocal images show an intact layer of hexagonal/polygonal cells in the cryopreserved hESC-derived CEC-injected monkey corneas (Figure 6A). The CEC density of the injected (right) eye was >70% of the CEC density of the untreated (left) eye, while the CEC density in the injected eyes of the injury-injection model monkeys is relatively lower compared with the injection model (>70% versus >80%), it remained stable (±6% and ±2% for M-4, and M-5, respectively) at the 3- and 18-month postinjection time points (Figure 6B). The pachymetry measurements confirmed that the central corneal thickness of the injected (right) eye remained comparable (±20 μm) to the untreated (left) eye at the 3- and 18-month postinjection time points (Figure 6C). IOP of the injected (right) and untreated (left) eyes remained within 9 to 14 mm Hg in both monkeys.

IHC analysis of human-specific nucleoli

The data presented in Figure S5 confirm that only hESC-derived CECs and not even derivatives of CECs injected into the anterior chamber can form a functional CE in the

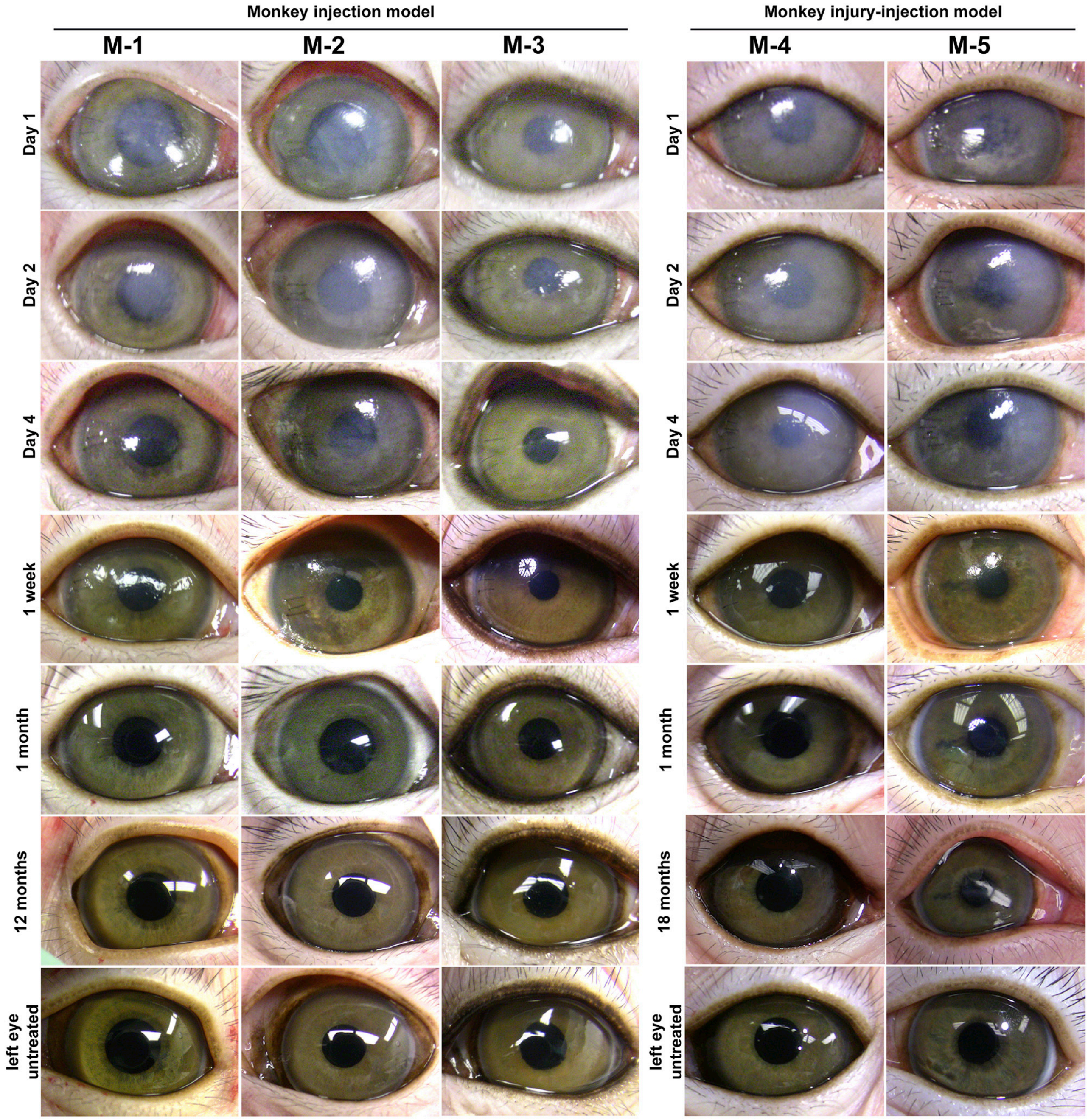

Figure 5. Illustration of corneal transparency after the injection of cryopreserved human embryonic stem cell (hESC)-derived corneal endothelial cells (CECs) in monkeys

Representative images of cryopreserved hESC-derived CEC-injected right eyes on days 1, 2, and 4, and 1 week, 1 month, and 12 months (M-1, M-2, and M-3) or 18 months (M-4 and M-5) postinjection along with untreated left eyes. The three monkeys (M-1, M-2, and M-3) represent the injection model (hESC-derived CECs injected immediately after the removal of the central corneal endothelium [CE]), and the two monkeys (M-4 and M-5) represent the injury-injection model (hESC-derived CECs injected 2 days after the removal of the central CE).

rabbit injection model. We planned to perform a similar experiment in the monkey injection model; however, since we were not able to devise a strategy to administer ointment on the corneal endothelial injury-induced cornea in monkeys multiple times in a 24-h period without sedating the animal, we, therefore, did not perform hESC-derived EnMT-CEC injection in monkeys. To confirm the human origin of regenerated CE in monkeys, we performed

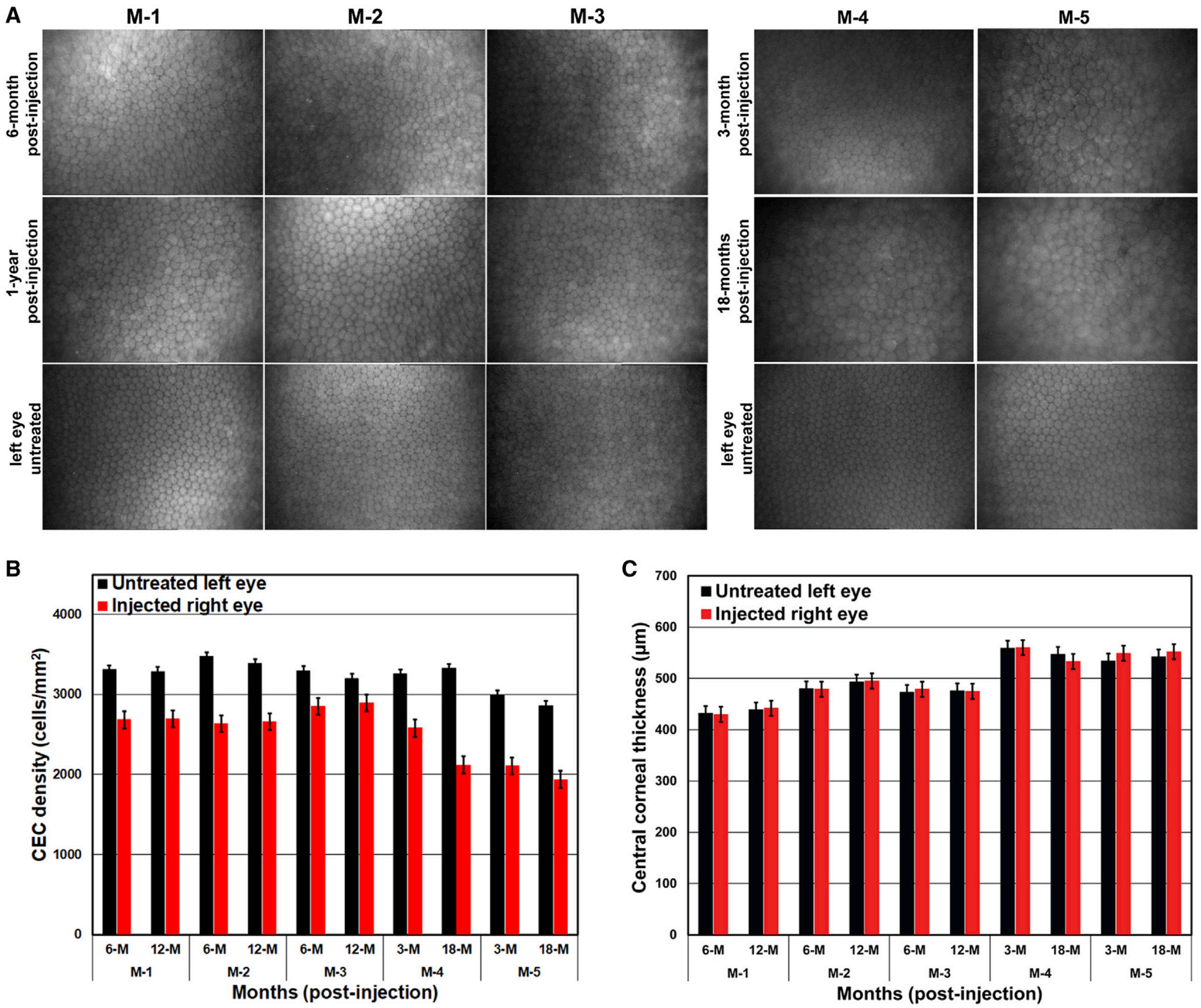

Figure 6. Evaluation of the regenerated corneal endothelium (CE) after injection of cryopreserved human embryonic stem cell (hESC)-derived corneal endothelial cells (CECs) in monkeys

The three monkeys (M-1, M-2, and M-3) represent the injection model (hESC-derived CECs injected immediately after the removal of the central CE), and the two monkeys (M-4 and M-5) represent the injury-injection model (hESC-derived CECs injected 2 days after the removal of the central CE).

(A) The CE of hESC-derived CEC-injected right eyes of M-1, M-2, and M-3 at 6 and 12 months postinjection and of M-4 and M-5 at 3 and 18 months postinjection along with untreated left eyes was examined using a confoscan4 scanning microscope.

(B) Illustration of the CEC density (cells/mm^2) of hESC-derived CEC-injected right eyes and untreated left eyes of M-1, M-2, and M-3 at 6 and 12 months postinjection, and of M-4 and M-5 at 3 and 18 months postinjection.

(C) Demonstration of the central corneal thickness of hESC-derived CEC-injected right eyes and untreated left eyes of M-1, M-2, and M-3 at 6 and 12 months postinjection, and of M-4 and M-5 at 3 and 18 months postinjection.

Note: Error bars represent standard deviation in each data set examined in four independent replicates.

immunostaining of human-specific nucleoli of formalin-fixed, paraffin-embedded sections of the injected right eye of the monkey injection model.

As shown in Figure S7, the IHC analysis of human-specific nucleoli demonstrates the human origin of CECs in the regenerated CE. It is worth noting that while the CE is regenerated by the injection of hESC-derived CECs, the corneal stroma consists of resident keratocytes. Importantly, the nuclei of these resident keratocytes (serving as an internal control) do not show immunoreactivity with the human-specific antibody. The IHC analysis confirms that the regenerated CE consists of hESC-derived CECs.

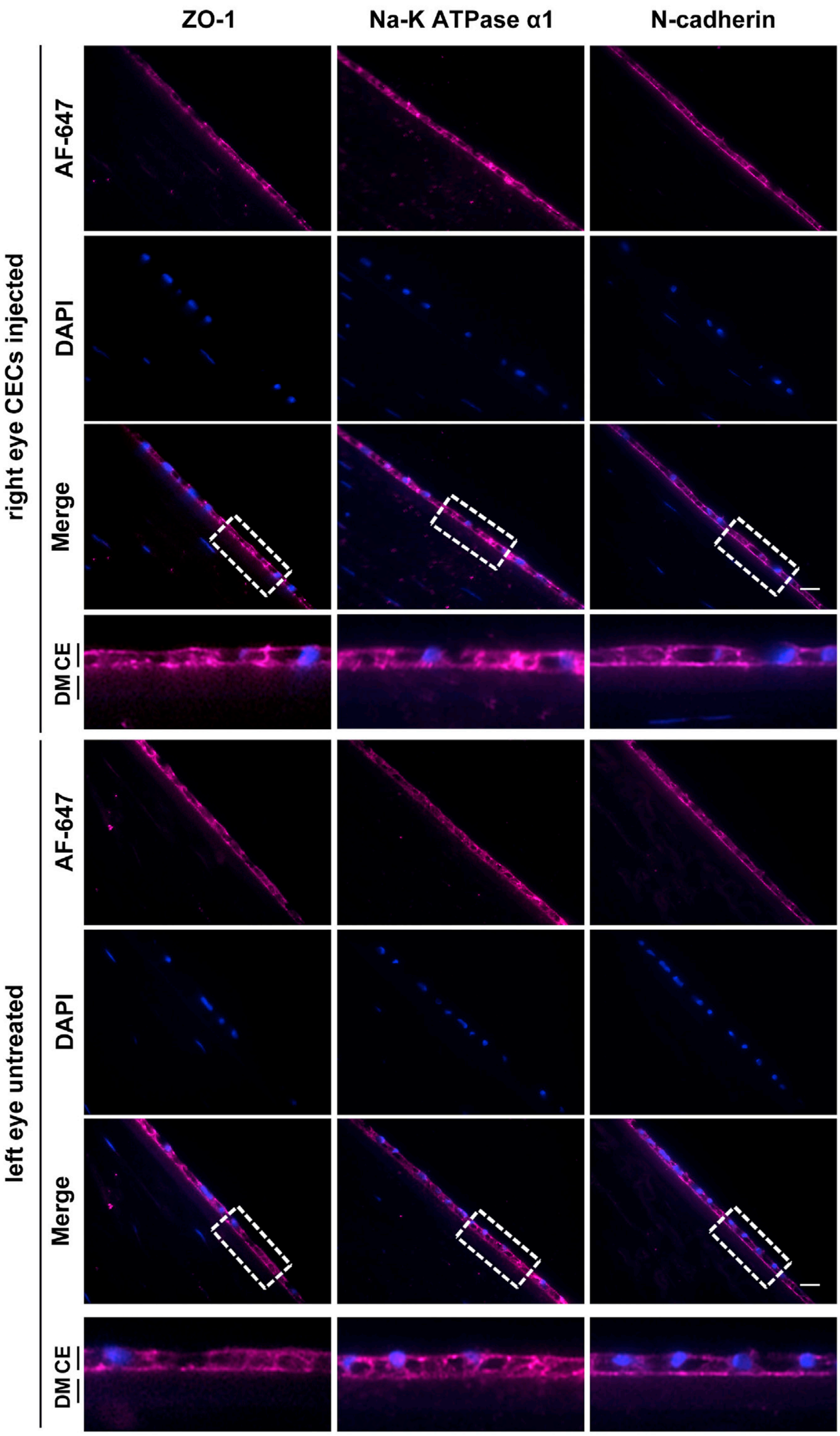

Figure 7. Immunohistochemical analysis of tight junction, pump function, and structural-integrity related proteins in the regenerated corneal endothelium (CE) in monkeys

The analysis of cryopreserved human embryonic stem cell (hESC)-derived corneal endothelial cell (CEC)-injected right eye and untreated left eye of monkey, M-2 representing the injection model (hESC-derived CECs injected immediately after the removal of the central corneal endothelium) after euthanization 12 months post-injection. Immunostaining of zona-occludens-1 (ZO-1), ATPase sodium/potassium subunit alpha1 (Na^+/K^+ ATPase α1), and N-cadherin was performed as described in experimental procedures. Cell nuclei were counterstained with DAPI (4′,6-diamidine-2′-phenylindole dihydrochloride). The boxed areas are enlarged and shown in the panels below. Note: The images were captured at ×60 magnification. Scale bar, 10 μm.

Necropsy of cryopreserved hESC-derived CEC-injected animals

The use of pluripotent stem cells and/or pluripotent stem cell derivatives raises reasonable concerns about teratoma formation. To address these concerns in rabbit models, we performed a CBC (complete blood count) analysis and a necropsy examination of five rabbits including (R-1, R-2, and R-3) 9 months postinjection (injection model) and two rabbits (R-4 and R-5) 18 months postinjection (injury-injection model). All parameters examined during

CBC analysis were within the normal physiological range. The necropsy examination of multiple tissues, including adrenal glands, gall bladder, brain, heart, kidneys, liver, lungs, optic nerves, pituitary glands, spleen, and urinary bladder, confirmed no abnormal pathology or teratomas observed in any of the five rabbits (Table S1).

To address the teratoma concerns in monkeys, we performed a CBC analysis of all five monkeys, and all parameters examined during analysis were within the normal physiological range. A necropsy examination of M-1, M-2 (12 months postinjection), M-3 (26 months postinjection), M-4, and M-5 (21 months postinjection) was completed examining multiple tissues, including adrenal glands, gall bladder, brain, heart, kidneys, liver, lungs, optic nerves, pituitary glands, spleen, and urinary bladder was completed. The outcome of the examination confirmed no abnormal pathology or teratomas observed in any of the five monkeys (Table S2).

DISCUSSION

Herein, we evaluate the efficacy of cryopreserved hESC-derived CECs to reconstitute a functional CE in rabbits and monkeys. We confirmed that cryopreservation does not affect the expression profile of hESC-derived CECs through next-generation RNA-Seq analysis. Next, we examined the potential of cryopreserved hESC-derived CECs to form a functional monolayer of CE in four different corneal endothelial dysfunction models in rabbits and monkeys. Our approach included an injection of 750,000 (in rabbits) and 1×10^6 (in monkeys) cryopreserved hESC-derived CECs into the anterior chamber either immediately after mechanical scraping of the central CE or a few days later when corneal edema developed. The eyes (both the injected right and the untreated left eye that served as a control) of all animals were monitored postinjection for corneal transparency, corneal thickness, CE cell density, and CE cell shape. IHC analysis was performed on injected and untreated eyes of both rabbits and monkeys, and CBC analysis and a necropsy examination was completed for all preclinical models.

The corneas of the injected eyes attained transparency within three weeks postinjection in all preclinical models (Figures 2 and 5). Confocal scanning microscopy confirmed an intact layer of hexagonal/polygonal cells in the CE formed from the injected cryopreserved hESC-derived CECs (Figures 3 and 6). The CEC count in the injected eye was >80% of the CEC count of the untreated control eye for all preclinical models except for the two injury-injection model monkeys, i.e., M4, and M5 where the CEC count in the injected eye was ~70% of the CEC count of the untreated control eye but remains stable 18 months postinjection (Figures 3 and 6). Pachymetry confirmed comparable (±20 μm) central corneal thickness of the injected and untreated eyes in all preclinical animal models (Figures 3 and 6). IHC analysis illustrated a monolayer of cells expressing barrier and pump function proteins in the regenerated CE (Figures 4 and 7). Finally, all parameters examined during the CBC analysis were within the normal physiological range and necropsy examination confirmed no remarkable change in multiple tissues examined for teratoma formation (Tables S1 and S2). Taken together, these data confirm that cryopreserved hESC-derived CECs can form a functional monolayer of CE on denuded DM even with corneal edema.

The CE maintains corneal transparency by mediating hydration through barrier and pump functions. The physiological functioning of the CE is substantially compromised with reduced CEC density resulting in corneal edema and loss of vision (Bonanno, 2012; Tan et al., 2012). In many areas around the world, there is a shortage of transplantable corneal tissue, which results in the inability to restore functional vision in countless patients affected with loss of vision due to corneal endothelial failure (Lehman et al., 2015; Mehta et al., 2008; Terry et al., 2008). Pluripotent stem cells, neural crest cells, skin-derived precursors, and corneal stromal stem cells, etc., have been explored for the possibility of providing functional CECs for the treatment of corneal endothelial dysfunction (Hatou et al., 2013; Ju et al., 2012; Shen et al., 2017; Song et al., 2016; Zhang et al., 2014).

Injection of cultured human CECs in combination with an ROCK inhibitor into the anterior chamber of the eye has been demonstrated to treat patients with corneal endothelial dystrophies including bullous keratopathy and Fuchs endothelial corneal dystrophy (Kinoshita et al., 2018). Kinoshita and colleagues injected human CECs that were cultured from donor corneas into the anterior chamber of the eye selected for treatment (Kinoshita et al., 2018). The authors confirm the CEC density exceeding 1,000 cells/mm^2 accompanied by a decrease in corneal thickness and an improvement in best-corrected visual acuity in the majority of treated eyes.

Our approach of delivering CECs is similar to that described by Kinoshita and colleagues (2018). The use of cultured human CECs likely will not alleviate the need for donor corneas. We believe that pluripotent stem cell–derived CECs may be an alternative to providing adequate quantities of CE worldwide to allow treatment for corneal endothelial failure–mediated vision loss. Both hESCs and the anterior segment of the eye have been reported of possessing immune-privileged characteristics (Li et al., 2004; Taylor, 2009), which provide an unprecedented opportunity for the use of hESC derivatives in translational research. In line with published reports, we observed no immune rejection resulting from the injection of cryopreserved hESC-derived CECs either in the rabbits and/or the monkeys during the postinjection periods.

The use of pluripotent stem cells and/or pluripotent stem cell derivatives raises reasonable concerns about teratoma formation. It is worth noting that our differentiation protocol consists of an intermediate differentiation step, i.e., differentiation of hESCs to neural crest cells before differentiation to CECs, which reduces the chance of having undifferentiated hESC in aliquots of hESC-derived CECs. In line with the advantage of having an intermediate differentiation step, we only identified residual levels of undifferentiated hESCs at day 20 by qRT-PCR, which is consistent with the results of the necropsy examination that confirmed no remarkable change in multiple tissues examined for teratoma formation.

Finally, the fate of the hESC-derived CECs that do not settle on the denuded DM and persist within the anterior chamber remains elusive. Most likely these unsettled cells pass through the trabecular meshwork without blocking the aqueous humor drainage and into the systemic circulation. Furthermore, we cannot rule out the possibility that these unsettled cells find their way to other body organs; however, it is worth noting that the necropsy examination of multiple tissues confirmed no remarkable pathology. In short, the precise whereabouts of the unsettled injected CECs remains unknown, and we plan to investigate the fate of the unsettled CECs through labeled pluripotent stem cell–derived CECs in future studies.

In conclusion, our data demonstrate the efficacy of cryopreserved hESC-derived CECs to form a functional CE on denuded DM when injected into the anterior chamber of preclinical models. Moreover, our data provide evidence suggestive of the clinical utility of pluripotent stem cell–derived CECs as an alternative to human donor CE in keratoplasty that will help combat the shortage of transplantable-grade corneal tissue, which, in turn, will help to reduce vision loss from CEC dysfunction. To the best of our knowledge, this is the first report confirming the efficacy of cryopreserved hESC-derived CECs as an alternative to donor CE in keratoplasty in multiple preclinical animal models.

EXPERIMENTAL PROCEDURES

Culturing of hESCs

H9 hESCs (WiCell Research Institute, Madison, WI) were cultured in Essential8 (E8) medium (Thermo Fisher Scientific; Waltham, MA) on Vitronectin (Thermo Fisher Scientific) coated plates under xeno-free conditions. The cells were passaged using 0.5 mM EDTA (Thermo Fisher Scientific) every 4 to 5 days, and the culture medium was changed daily.

Generation of hESC-derived CECs under xeno-free conditions

H9 hESCs were used to generate CECs under xeno-free conditions by modifying our published procedure (Ali et al., 2018a). Briefly, hESCs were seeded on 35 mm Vitronectin (Thermo Fisher Scientific) coated plates in 1:7 dilutions (85% confluent plate was split into seven plates) on day 0 using 0.5 mM EDTA (Thermo Fisher Scientific). The hESCs were grown for 4 days in E8 media. On day 4, E8 media was replaced with dual Smad inhibitors media containing 500 ng/mL recombinant human Noggin (R&D Systems, Minneapolis, MN) and 10 μM SB431542 (MilliporeSigma, Burlington, MA) in a basal media of 80% DMEM/F-12 (Thermo Fisher Scientific), 20% knockout serum replacement (KSR; Thermo Fisher Scientific), 1% nonessential amino acids (Thermo Fisher Scientific), 1 mM L-glutamine (Thermo Fisher Scientific), 0.1 mM β-mercaptoethanol (MilliporeSigma), and 8 ng/mL basic fibroblast growth factor (bFGF; R&D Systems).

On day 6, dual Smad inhibitor media was replaced by cornea medium containing 0.1× B27 supplement (Thermo Fisher Scientific), 10 ng/mL recombinant human platelet-derived growth factor-BB (PDGF-BB; PeproTech, Rocky Hill, NJ), and 10 ng/mL recombinant human Dickkopf related protein-2 (DKK-2; R&D Systems) in a basal media of 80% DMEM/F-12 (Thermo Fisher Scientific), 20% KSR (Thermo Fisher Scientific), 1% nonessential amino acids (Thermo Fisher Scientific), 1 mM L-glutamine (Thermo Fisher Scientific), 0.1 mM β-mercaptoethanol (MilliporeSigma), and 8 ng/mL bFGF (R&D Systems). On day 7, the differentiating CECs were transferred to new Vitronectin-coated plates (35 mm) and were grown in cornea medium for 13 additional days. The differentiated CECs at day 20 were characterized by phase-contrast microscopy, immunocytochemistry, qRT-PCR and next-generation RNA-Seq.

Cryopreservation of hESC-derived CECs

The hESC-derived CECs were detached using cell dissociation buffer (Thermo Fisher Scientific) and cryopreserved in CryoStor cell cryopreservation media (MilliporeSigma). The cryopreserved hESC-derived CECs were stored in the liquid nitrogen for 3 to 6 weeks and used for qRT-PCR, RNA-Seq analysis, and injection in rabbits and monkeys. The hESC-derived CEC vials were removed from liquid nitrogen to prepare cells for the injection. The hESC-derived CECs were first washed with DMEM/F-12 medium supplemented with 20% KSR and washed twice with serum-free DMEM/F-12. The cell count was determined using an automated cell counter (Countess; Invitrogen). A total of 7.5×10^5 cells were suspended in 200 μL of DMEM/F-12 medium supplemented with 100 μM Rock inhibitor Y-27632 for injection in rabbits, whereas 1.0×10^6 cells were suspended in 250 μL of DMEM/F-12 medium supplemented with 100 μM Rock inhibitor Y-27632 for injection in monkeys.

Generation of hESC-derived EnMT-CECs

The hESC-derived CECs were transformed into EnMT-CECs according to published protocol with brief modifications (Yamashita et al., 2018). To generate hESC-derived EnMT-CECs, the hESC-derived CECs at day 20 were reseeded on 35-mm Vitronectin-coated plates in 80% DMEM/F-12 (Thermo Fisher Scientific) supplemented with 20% KSR, 1% Insulin, Transferrin, Selenium solution (ITS-G; MilliporeSigma), 1 ng/mL transforming growth factor β1 (TGFβ1; R&D systems), 2 mM glutamate (Thermo Fisher Scientific), and 20 ng/mL of bFGF (R&D Systems). The culture medium was changed daily, the prospective hESC-derived EnMT-

CECs were passaged until passage 4, characterized by phase-contrast microscopy and cryopreserved in CryoStor cell cryopreservation media.

Institutional approvals and study design

Male and female New Zealand white rabbits 12 to 14 weeks old weighing 2.5 to 3.5 kg were purchased from Charles River Laboratories (Charles River Laboratories, Wilmington, MA) while the male rhesus monkeys, approximately 14 years old, weighing 11 to 14 kg were obtained from the Johns Hopkins Research Animal Resources (Baltimore, MD). The use of animals (rabbit and monkey) was approved by the ACUC of Johns Hopkins University School of Medicine consistent with the Association of Research in Vision and Ophthalmology (ARVO) statement for the use of animals in ophthalmic and vision research and all experiments were performed in accordance with the approved protocols.

In the rabbit study, rabbits were divided into two groups for injection of cryopreserved hESC-derived CECs. First, the injection model where cryopreserved hESC-derived CECs were injected immediately after the removal of the central CE (n = 3), and second, the injury-injection model where cryopreserved hESC-derived CECs were injected 4 days after the removal of the central CE (n = 2). In addition to the two groups, rabbits (n = 3) were injected with cryopreserved hESC-derived EnMT-CECs following the injection model, i.e., hESC-derived EnMT-CECs injected immediately after the removal of the central CE.

The monkeys were also divided into two groups for injection of cryopreserved hESC-derived CECs. First, the injection model where cryopreserved hESC-derived CECs were injected immediately after the removal of the central CE (n = 3), and second, the injury-injection model where cryopreserved hESC-derived CECs were injected 2 days after the removal of the central CE (n = 2).

Injection model in rabbits

To inject cryopreserved hESC-derived CECs (injection model), rabbits were anesthetized using acepromazine (subcutaneous), dexmedetomidine (intramuscular), and ketamine (intramuscular). The rabbits were also given buprenorphine (subcutaneous), dexamethasone (intramuscular), and kenalog-40 (intramuscular) to inhibit pain, inflammation, and immunologic reaction. Tracheal intubation was performed to enable inhalation of isoflurane and oxygen mixture during the surgical procedure for sedation. Rabbits were also injected with lactated Ringer's solution at a rate of 15 mL per hour in the right ear using a 22-gauge catheter during the entire surgical procedure. Pupils were dilated using a 2.5% phenylephrine solution and 1% tropicamide solution and followed by the application of 0.5% proparacaine hydrochloride ophthalmic solution.

The central CE, consisting of 8-mm diameter was marked and a 1.6-mm incision was made at the temporal limbus. A 20-gauge silicone needle (Inami & Co., Ltd., Tokyo, Japan) was used to scrape resident CECs within the marked 8-mm central CE. It is noteworthy that while 8 mm of the central CE is targeted, it is difficult to avoid the removal of some CECs residing in the immediate proximity outside the marked CE. A 30-gauge cannula was used to irrigate the anterior chamber to remove the cell debris from the anterior chamber. A 28-gauge needle was employed to inject 7.5×10^5 cryopreserved hESC-derived CECs in 200 μL of DMEM/F-12 medium supplemented with 100 μM of ROCK inhibitor Y-27632 into the anterior chamber. The incision was closed by three interrupted sutures (Nylon 10-0; Mani, Inc. Tochigi, Japan). The rabbits were then immediately placed in an eye-down position for 3 h to allow the injected cells to settle on denuded DM. After 3 h, the middle suture was removed, and the anterior chamber was washed twice with sterile balanced salt solution (BSS) to remove unsettled cells. Finally, the anterior chamber was reconstituted with BSS and the incision was closed using one interrupted suture (Nylon 10-0; Mani, Inc.).

Tobradex ophthalmic ointment was applied three times a day for 10 days postinjection. In the preliminary experiments, we confirmed that the DM remains intact, and the mechanically scraped area was devoid of cells on the DM by H&E and Calcein-AM staining, respectively.

Clinical evaluation of rabbit eyes

The clinical evaluation of CEC-injected and untreated control eyes was performed by evaluating IOP, corneal thickness, and CEC density using Tono-Pen XL (iKiss, Scottsdale, AZ), ultrasonic pachymeter (Accutome Inc., Malvern, PA), and confoscan4 scanning microscope (Nidek Technologies), respectively at different time points. The images of the injected corneas were recorded for each time point.

Histological evaluation of rabbit eyes

The injection model rabbits were euthanized by an intravenous injection of euthanasia solution 9 months postinjection while the cryopreserved hESC-derived EnMT-CEC-injected rabbits were euthanized 28 days postinjection. The injected and control eyes were removed, fixed in 10% formalin, and subjected to H&E staining and IHC analysis. H&E staining was completed by subjecting the sections to deparaffinization and hydration, followed by staining with hematoxylin and counterstaining with eosin, and finally dehydration and mounting.

IHC analysis was performed by subjecting the sections to deparaffinization, hydration, and antigen retrieval followed by blocking with 5% BSA (MilliporeSigma) for 45 min at room temperature. The sections were first incubated 1:300 ZO-1 (catalog # MABT339; MilliporeSigma), 1:100 Na^+/K^+ ATPase α1 (catalog # MA1-16731; Thermo Fisher Scientific), and 1:250 N-cadherin (catalog # 33–3900; Thermo Fisher Scientific) primary antibodies overnight at 4°C. The sections were next treated with 1:100 FITC-conjugated goat anti-mouse immunoglobulin (Ig)G (for ZO-1; catalog # AP124F; MilliporeSigma), 1:200 Alexa Fluor 647 donkey anti-mouse IgG (for Na^+/K^+ ATPase α1; catalog # A-31571; Thermo Fisher Scientific), and 1:100 FITC-conjugated goat anti-mouse IgG (for N-cadherin; catalog # AP124F; MilliporeSigma) secondary antibodies for 2 h at room temperature. The nuclei were counterstained with DAPI (4′,6-diamidine-2′-phenylindole dihydrochloride; MilliporeSigma).

A total of five random field images per section were captured for the injected area with at least two sections from each eye using a microscope (Olympus LX81; Olympus, Tokyo, Japan) equipped with software (Slidebook Software 3i; Denver, CO) and prepared using image editing software (Adobe Photoshop CS5; Adobe Systems, Inc., San Jose, CA).

hESC-derived EnMT-CEC injection in rabbits

A total of 750,000 cryopreserved hESC-derived EnMT-CECs in 200 μL of DMEM/F-12 medium supplemented with 100 μM of ROCK inhibitor Y-27632 were injected following corneal endothelial injury according to the procedure described in the injection model in rabbits. The hESC-derived EnMT-CEC-injected rabbits were housed for 28 days postinjection and euthanized afterward.

Injury-injection model in rabbits

The procedure for injecting the cryopreserved hESC-derived CECs in injury-injection model rabbits is similar to the procedure described above for the rabbit injection model with the exception that after removal of the central CE, the rabbits were housed for 4 days with topical application of Tobradex ophthalmic ointment three times a day. On day 4 postremoval of the central resident CECs, the sutures were removed, and cellular debris was scraped using the 20-gauge silicone needle followed by washing of the anterior chamber with sterile BSS and injection of 7.5×10^5 cryopreserved hESC-derived CECs in 200 μL of DMEM/F-12 medium supplemented with 100 μM of ROCK inhibitor Y-27632 into the anterior chamber.

Injection model in monkeys

To inject cryopreserved hESC-derived CECs (injection model), monkeys were sedated with ketamine and dexmedetomidine. Tracheal intubation was performed to enable inhalation of isoflurane and oxygen mixture during the surgical procedure for sedation. Pupils were dilated using a 2.5% phenylephrine solution and 1% tropicamide solution and followed by the application of 0.5% proparacaine hydrochloride ophthalmic solution.

The central CE consisting of 8-mm diameter was marked and a 1.6-mm incision was made at the temporal limbus. A 20-gauge silicone needle (Inami & Co., Ltd., Tokyo, Japan) was used to scrape resident CECs within the marked 8-mm central CE. As mentioned above, although 8 mm of the central CE is targeted, it is difficult to avoid the removal of some CECs residing in the immediate proximity outside the marked CE. A 30-gauge cannula was used to irrigate the anterior chamber to remove the cell debris from the anterior chamber. A 28-gauge needle was employed to inject 1.0×10^6 cryopreserved hESC-derived CECs in 250 μL of DMEM/F-12 medium supplemented with 100 μM of ROCK inhibitor Y-27632 into the anterior chamber. The incision was closed by three interrupted sutures (Nylon 10-0; Mani, Inc., Tochigi, Japan). The animal was immediately placed in an eye-down position for 3 h to allow the injected cells to settle on denuded DM. After 3 h, the middle suture was removed, the anterior chamber was washed twice with sterile BSS to remove unsettled cells. Finally, the anterior chamber was reconstituted with BSS and the incision was closed using one interrupted suture (Nylon 10-0; Mani, Inc.).

Tobradex ophthalmic ointment and kenalog-40 (to inhibit inflammation and immunologic rejection) were administered every alternate day for 12 days postinjection using dexmedetomidine and ketamine sedatives.

Clinical evaluation of monkey eyes

The clinical evaluation of the injected and control eyes was performed by evaluating IOP, corneal thickness, and CEC density using Tono-Pen XL (iKiss), ultrasonic pachymeter (Accutome Inc.), and confoscan4 scanning microscope (Nidek Technologies), respectively, at different time points. The images of the injected corneas were recorded for each time point.

Histological evaluation of monkey eyes

Monkeys were sedated using dexmedetomidine and ketamine and euthanized by an intravenous injection of the euthanasia solution 12 months postinjection (or 26 months postinjection for M-3). The injected and control eyes were removed, fixed in 10% formalin, and subjected to H&E staining and IHC analysis. H&E staining was completed by subjecting the sections to deparaffinization and hydration, followed by staining with hematoxylin and counterstaining with eosin, and finally dehydration and mounting.

IHC analysis was performed by subjecting the sections to deparaffinization, hydration, and antigen retrieval followed by blocking with 5% BSA (MilliporeSigma) for 45 min at room temperature. The sections were first incubated 1:300 ZO-1 (catalog #MABT339; MilliporeSigma), 1:100 Na^+/K^+ ATPase α1 (catalog # MA1-16731; Thermo Fisher Scientific), and 1:250 N-cadherin (catalog # 33–3900; Thermo Fisher Scientific) primary antibodies overnight at 4°C. The sections were next treated with 1:200 Alexa Fluor 647 donkey anti-mouse IgG (catalog #A-31571; Thermo Fisher Scientific) for ZO-1, Na^+/K^+ ATPase α1, and N-cadherin secondary antibody for 2 h at room temperature. The nuclei were counterstained with DAPI.

In addition, to confirm the human origin of regenerated CE from injected cryopreserved hESC-derived CECs into the monkey eye, the sections were first incubated with 1:200 human-specific nucleoli (catalog #ab190710; Abcam) primary antibody overnight at 4°C and subsequently treated with 1:200 Alexa Fluor 647 goat anti-mouse IgG (catalog #ab150115; Abcam) secondary antibody for 2 h at room temperature while rest of the IHC procedure followed was similar to described above. Imaging of regenerated CE from injected hESC-derived CECs was performed using a microscope (Zeiss LSM710; Carl Zeiss, Jena, Germany).

A total of five random field images per section were captured for the injected area with at least two sections from each eye and prepared using image editing software (Adobe Photoshop CS5; Adobe Systems, Inc., San Jose, CA).

Injury-injection model in monkeys

To inject cryopreserved hESC-derived CECs (injury-injection model), following the removal of 8-mm central resident CECs, using a 20-gauge silicone needle (as performed above in the injection model), the anterior chamber was reconstituted with sterile BSS and the incision was closed using 10-0 Nylon suture (Mani, Inc.). The procedure for injecting the cryopreserved hESC-derived CECs in injury-injection model monkeys is similar to the procedure described above for the monkey injection model with the exception that after removal of the central CE, the monkeys were housed for 2 days with the topical application of Tobradex ophthalmic ointment after every 24 h. On day 2 postremoval of the central CECs, the sutures were removed, and cellular debris was removed using the 20-gauge silicone needle followed by washing of the anterior chamber with sterile BSS and injection of

1.0×10^6 cryopreserved hESC-derived CECs in 250 μL of DMEM/F-12 medium supplemented with 100 μM of ROCK inhibitor Y-27632 into the anterior chamber.

Necropsy and CBC examination

The injection model rabbits were housed for 9 months and the injury-injection model rabbits were housed 18 months postinjection before they were euthanized. The injection model monkeys were housed for 12 months (except for M3 that was housed for 26 months) and injury-injection model monkeys were housed for 21 months postinjection prior to euthanization. Both rabbits and monkeys were subjected to a CBC analysis and necropsy examination. The tissues examined included adrenal glands, brain, femur with bone marrow, gall bladder, heart, kidneys, liver, lungs, optic nerves, pituitary glands, spleen, and urinary bladder.

Data and code availability

Next-generation RNA-Seq data reported in this manuscript are accessible through GEO accession number GSE173692.

SUPPLEMENTAL INFORMATION

Supplemental information can be found online at https://doi.org/10.1016/j.stemcr.2021.07.008.

AUTHOR CONTRIBUTIONS

M.A. and S.A.R. conceived and designed the experiments; M.A., S.Y.K., A.K., and S.A.R. were involved in culturing of pluripotent stem cells and differentiation to CECs; M.A. and S.A.R. contributed to the cryopreservation of CECs and preparation of CEC injection; M.A., S.Y.K., E.K.H., and S.A.R. contributed to the writing of the protocols for the use of animals in vision research; M.A., S.Y.K., and S.A.R. are personnel included on the rabbit and monkey protocols; M.A., S.Y.K., and S.A.R. performed surgical and clinical procedures including corneal endothelium scraping, and CEC injection; M.A., S.Y.K., and S.A.R. performed postinjection treatments and ascertained postinjection clinical data; M.A., S.Y.K., and S.A.R. completed histology and immunohistochemical examination; M.A., S.Y.K., J.D.G., and S.A.R. completed the clinical assessments; M.A., S.Y.K., J.D.G., E.K.H., A.K., and S.A.R. contributed to the writing of the manuscript.

ACKNOWLEDGMENTS

The research work reported in this study was supported by the Maryland Stem Cell Research Fund Grant 2020-MSCRFV-5241 (SAR).

Received: January 8, 2021
Revised: July 7, 2021
Accepted: July 7, 2021
Published: August 5, 2021

REFERENCES

Ali, M., Khan, S.Y., Vasanth, S., Ahmed, M.R., Chen, R., Na, C.H., Thomson, J.J., Qiu, C., Gottsch, J.D., and Riazuddin, S.A. (2018a). Generation and proteome profiling of PBMC-originated, iPSC-derived corneal endothelial cells. Invest Ophthalmol. Vis. Sci. *59*, 2437–2444.

Ali, M., Khan, S.Y., Kabir, F., Gottsch, J.D., and Riazuddin, S.A. (2018b). Comparative transcriptome analysis of hESC- and iPSC-derived corneal endothelial cells. Exp. Eye Res. *176*, 252–257.

Bonanno, J.A. (2012). Molecular mechanisms underlying the corneal endothelial pump. Exp. Eye Res. *95*, 2–7.

Gain, P., Jullienne, R., He, Z., Aldossary, M., Acquart, S., Cognasse, F., and Thuret, G. (2016). Global survey of corneal transplantation and eye banking. JAMA Ophthalmol. *134*, 167–173.

Hatou, S., Yoshida, S., Higa, K., Miyashita, H., Inagaki, E., Okano, H., Tsubota, K., and Shimmura, S. (2013). Functional corneal endothelium derived from corneal stroma stem cells of neural crest origin by retinoic acid and Wnt/beta-catenin signaling. Stem Cells Dev. *22*, 828–839.

Ju, C., Zhang, K., and Wu, X. (2012). Derivation of corneal endothelial cell-like cells from rat neural crest cells in vitro. PLoS One *7*, e42378.

Kinoshita, S., Koizumi, N., Ueno, M., Okumura, N., Imai, K., Tanaka, H., Yamamoto, Y., Nakamura, T., Inatomi, T., Bush, J., et al. (2018). Injection of cultured cells with a ROCK inhibitor for bullous keratopathy. N. Engl. J. Med. *378*, 995–1003.

Lehman, R.E., Copeland, L.A., Stock, E.M., and Fulcher, S.F. (2015). Graft detachment rate in DSEK/DSAEK after same-day complete air removal. Cornea *34*, 1358–1361.

Li, L., Baroja, M.L., Majumdar, A., Chadwick, K., Rouleau, A., Gallacher, L., Ferber, I., Lebkowski, J., Martin, T., Madrenas, J., et al. (2004). Human embryonic stem cells possess immune-privileged properties. Stem Cells *22*, 448–456.

McCabe, K.L., Kunzevitzky, N.J., Chiswell, B.P., Xia, X., Goldberg, J.L., and Lanza, R. (2015). Efficient generation of human embryonic stem cell-derived corneal endothelial cells by directed differentiation. PLoS One *10*, e0145266.

Mehta, J.S., Chua, J., Poh, R., Beuerman, R.W., and Tan, D. (2008). Primary graft failure after Descemet-stripping automated endothelial keratoplasty: clinico-pathological study. Cornea *27*, 722–726.

Okumura, N., Sakamoto, Y., Fujii, K., Kitano, J., Nakano, S., Tsujimoto, Y., Nakamura, S., Ueno, M., Hagiya, M., Hamuro, J., et al. (2016). Rho kinase inhibitor enables cell-based therapy for corneal endothelial dysfunction. Sci. Rep. *6*, 26113.

Peh, G.S.L., Ong, H.S., Adnan, K., Ang, H.P., Lwin, C.N., Seah, X.Y., Lin, S.J., and Mehta, J.S. (2019). Functional evaluation of two corneal endothelial cell-based therapies: tissue-engineered construct and cell injection. Sci. Rep. *9*, 6087.

Shen, L., Sun, P., Zhang, C., Yang, L., Du, L., and Wu, X. (2017). Therapy of corneal endothelial dysfunction with corneal endothelial cell-like cells derived from skin-derived precursors. Sci. Rep. *7*, 13400.

Song, Q., Yuan, S., An, Q., Chen, Y., Mao, F.F., Liu, Y., Liu, Q., and Fan, G. (2016). Directed differentiation of human embryonic stem cells to corneal endothelial cell-like cells: a transcriptomic analysis. Exp. Eye Res. *151*, 107–114.

Tan, D.T., Dart, J.K., Holland, E.J., and Kinoshita, S. (2012). Corneal transplantation. Lancet *379*, 1749–1761.

Taylor, A.W. (2009). Ocular immune privilege. Eye (Lond) *23*, 1885–1889.

Terry, M.A., Chen, E.S., Shamie, N., Hoar, K.L., and Friend, D.J. (2008). Endothelial cell loss after Descemet's stripping endothelial keratoplasty in a large prospective series. Ophthalmology *115*, 488–496.

Van Horn, D.L., Sendele, D.D., Seideman, S., and Buco, P.J. (1977). Regenerative capacity of the corneal endothelium in rabbit and cat. Invest Ophthalmol. Vis. Sci. *16*, 597–613.

Wagoner, M.D., Bohrer, L.R., Aldrich, B.T., Greiner, M.A., Mullins, R.F., Worthington, K.S., Tucker, B.A., and Wiley, L.A. (2018). Feeder-free differentiation of cells exhibiting characteristics of corneal endothelium from human induced pluripotent stem cells. Biol. Open. *7*, bio032102.

Yamashita, K., Hatou, S., Inagaki, E., Higa, K., Tsubota, K., and Shimmura, S. (2018). A rabbit corneal endothelial dysfunction model using endothelial-mesenchymal transformed cells. Sci. Rep. *8*, 16868.

Zhang, K., Pang, K., and Wu, X. (2014). Isolation and transplantation of corneal endothelial cell-like cells derived from in-vitro-differentiated human embryonic stem cells. Stem Cells Dev. *23*, 1340–1354.

Zhao, J.J., and Afshari, N.A. (2016). Generation of human corneal endothelial cells via in vitro ocular lineage restriction of pluripotent stem cells. Invest Ophthalmol. Vis. Sci. *57*, 6878–6884.

Stem Cell Reports, Volume *16*

Supplemental Information

Pluripotent stem cell–derived corneal endothelial cells as an alternative to donor corneal endothelium in keratoplasty

Muhammad Ali, Shahid Y. Khan, John D. Gottsch, Eric K. Hutchinson, Aisha Khan, and S. Amer Riazuddin

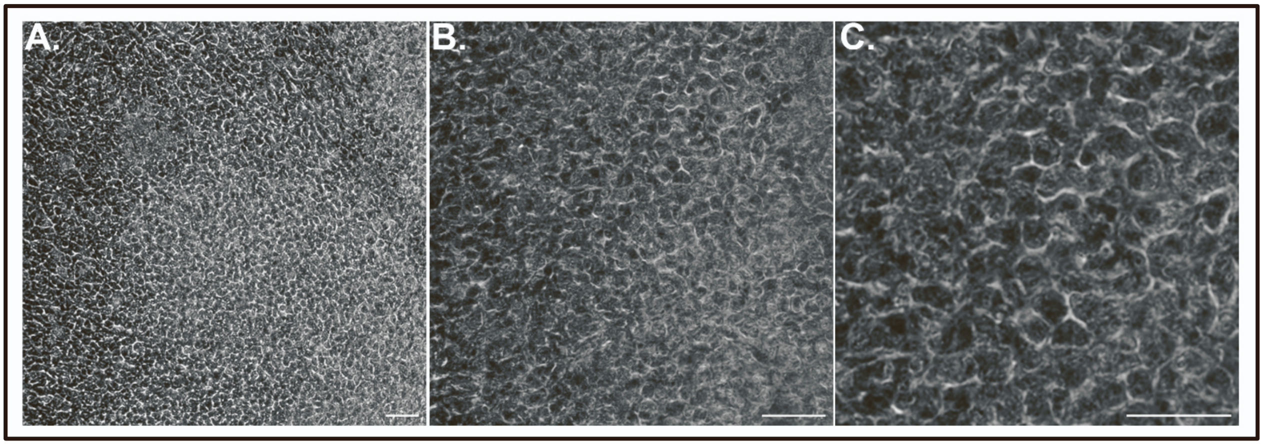

Supplementary Figure 1. Phase-contrast microscopy of human embryonic stem cell-derived corneal endothelial cells

The images of cells at differentiation day 20 are captured at (A) 10x, (B) 20x, and (C) 40x magnifications. Scale bars, 50 μm.

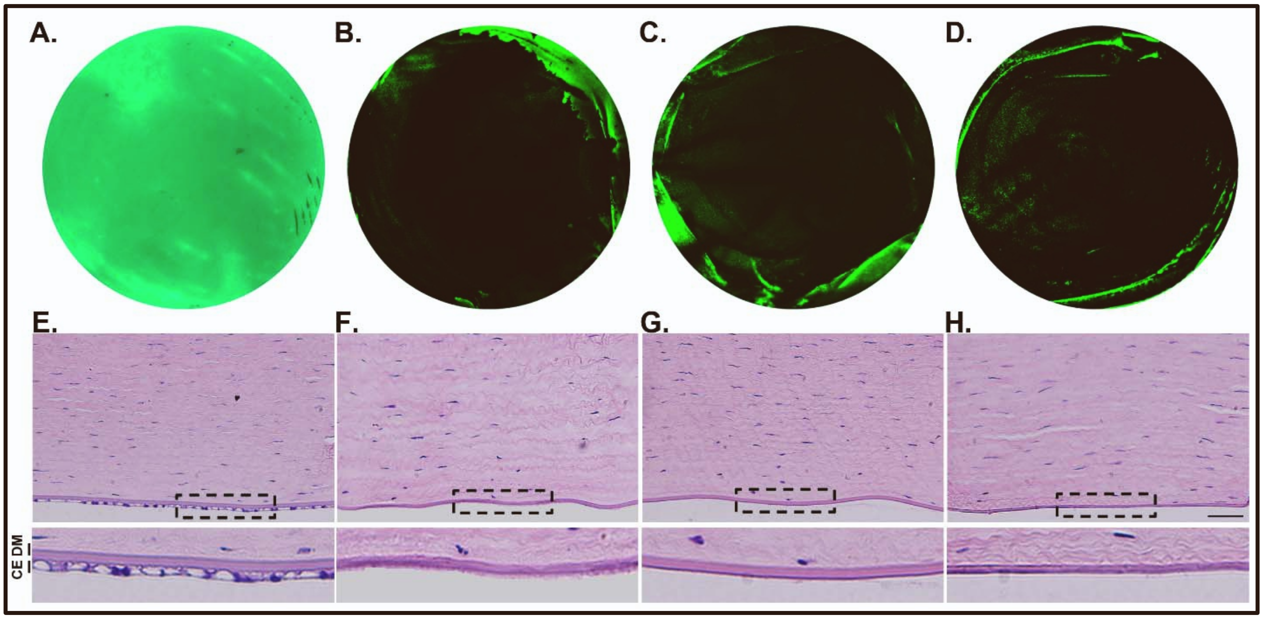

Supplementary Figure 2. Evaluation of the removal of corneal endothelium (CE) in rabbits by Calcein-AM and hematoxylin and eosin (H&E) staining

(A) An untreated control rabbit eye illustrating Calcein-AM stained intact CE. (B-D) The absence of staining confirms that the scrapped area is devoid of corneal endothelial cells (CECs) following the mechanical scraping of the CE. (E) H&E stained untreated control rabbit eye with an intact CE adherent to the Descemet's membrane (DM). (F-H) The scrapped area is devoid of CECs with an intact DM following the mechanical scraping of CE. The boxed areas are enlarged and shown in panels below. The images are captured at 20x magnification. Scale bar, 50 µm.

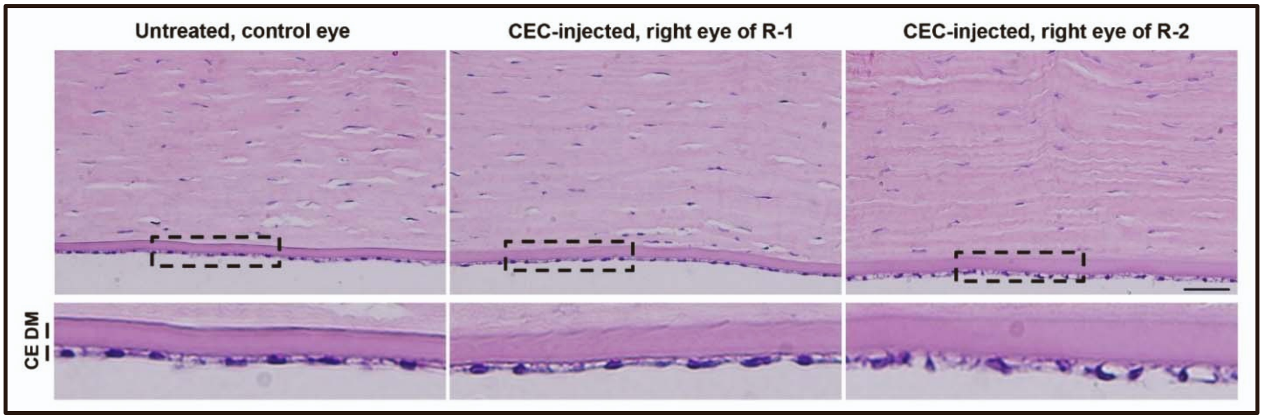

Supplementary Figure 3. Hematoxylin and eosin (H&E) staining of cryopreserved human embryonic stem cell (hESC)-derived corneal endothelial cell (CEC) injected rabbit corneas

Rabbit corneas of untreated control eye and cryopreserved hESC-derived CEC injected right eyes of R-1 and R-2. The rabbits (R-1, and R-2) represent the injection model where hESC-derived CECs are injected immediately after the removal of the central corneal endothelium. The boxed areas are enlarged and shown in panels below. The images are captured at 20x magnification. Scale bar, 50 µm.

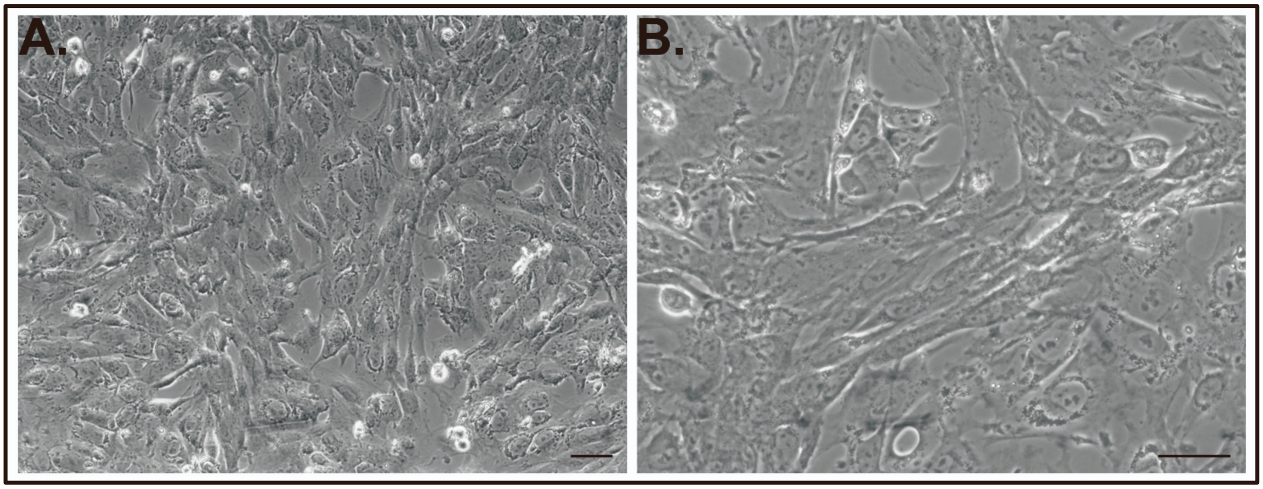

Supplementary Figure 4. Phase-contrast microscopy of human embryonic stem cell-derived endothelial mesenchymal transformed corneal endothelial cells

The images of cells at passage 4 are captured at (A) 10x, and (B) 20x magnification. Scale bars, 50 μm.

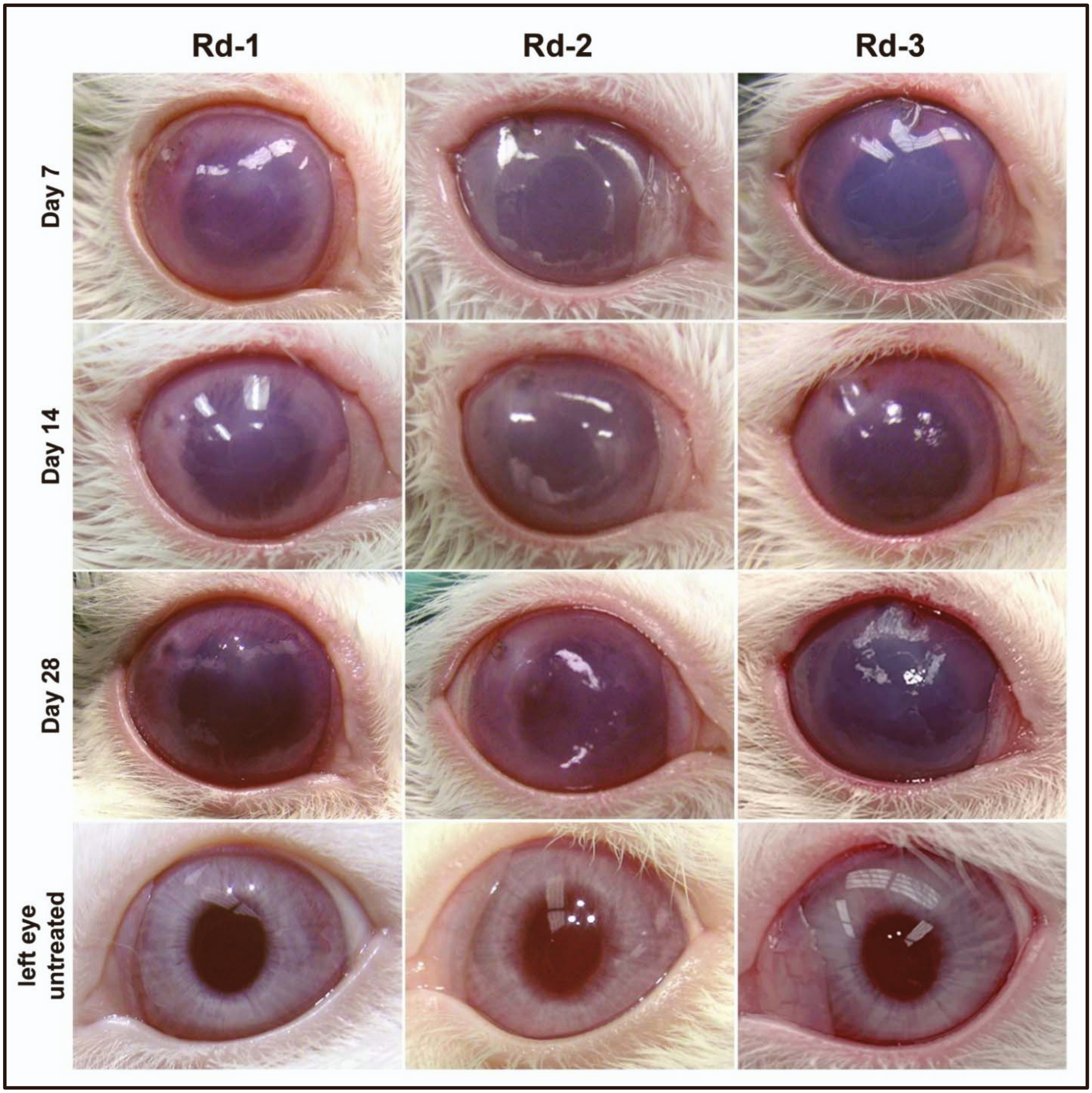

Supplementary Figure 5. Illustration of lack of corneal transparency after injection of cryopreserved human embryonic stem cell-derived endothelial mesenchymal transformed corneal endothelial cells (hESC-derived EnMT-CECs) in rabbits

Representative images of the cryopreserved hESC-derived EnMT-CEC injected right eyes on days 7, 14, and 28, and untreated left eyes.

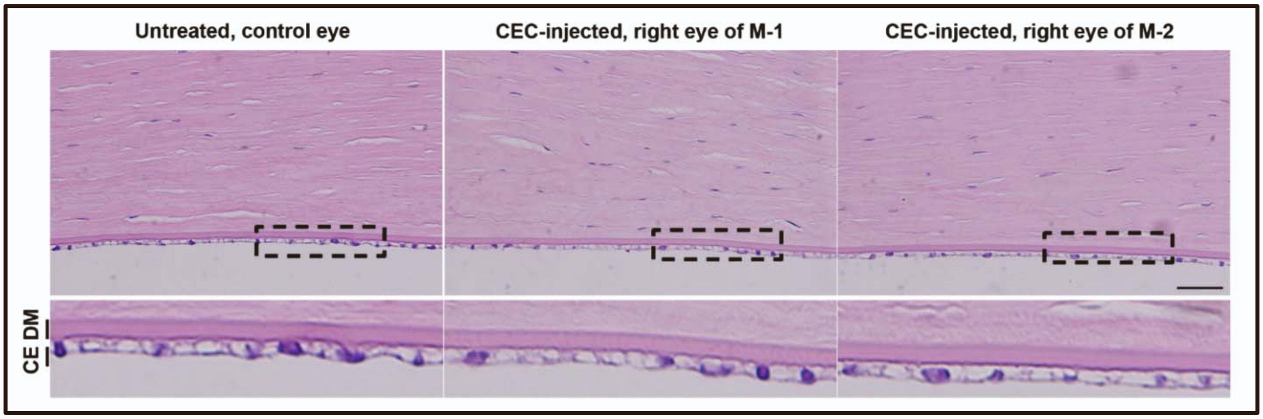

Supplementary Figure 6. Hematoxylin and eosin (H&E) staining of cryopreserved human embryonic stem cell (hESC)-derived corneal endothelial cell (CEC) injected monkey corneas

Monkey corneas of untreated control eye and cryopreserved hESC-derived CEC injected right eyes of M-1 and M-2. The monkeys (M-1, and M-2) represent the injection model where hESC-derived CECs were injected immediately after the removal of the central corneal endothelium. The boxed areas are enlarged and shown in panels below. The images are captured at 20x magnification. Scale bar, 50 μm.

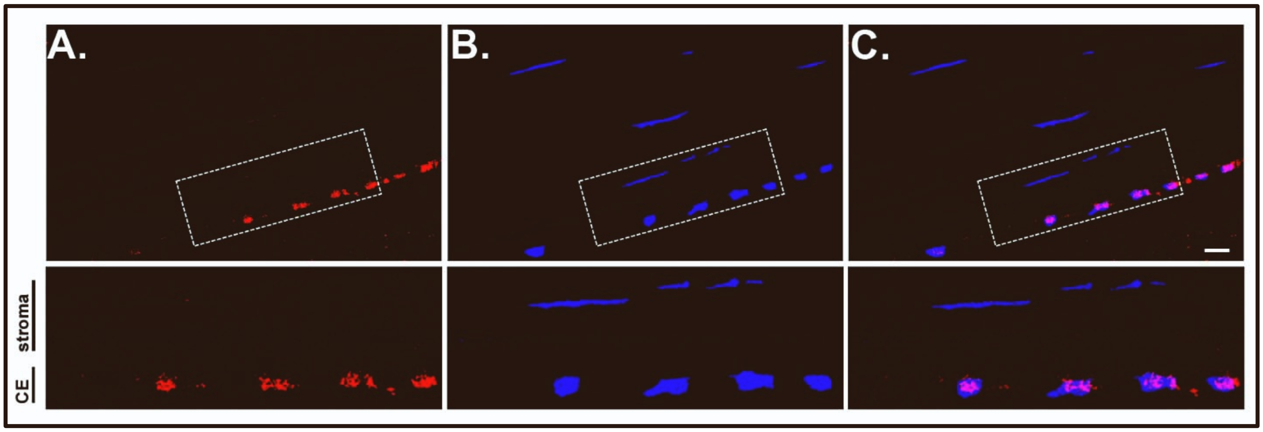

Supplementary Figure 7. Immunohistochemical analysis of the human antigen in cryopreserved human embryonic stem cell (hESC)-derived corneal endothelial cell (CEC) injected monkey corneas

The analysis confirms that the regenerated corneal endothelium (CE) of the right eye of the monkey (M-1) consists of cryopreserved hESC-derived CECs. (A) Immunostaining of human-specific nucleoli antibody. (B) Cell nuclei are counterstained with DAPI (4′,6-diamidine-2′-phenylindole dihydrochloride). (C) Merge of images shown in a and b. The corneal stroma consists of resident keratocytes, which serve as an internal negative control, and importantly, do not show reactivity with the human-specific antibody. M-1 represents the injection model monkey where hESC-derived CECs were injected immediately after the removal of the central CE. The boxed areas are enlarged and shown in panels below. The images are captured at 40x magnification. Scale bar, 10 μm.

Supplementary Table 1: The summary of the necropsy examination of rabbits injected with cryopreserved human embryonic stem cell (hESC)-derived corneal endothelial cells (CECs)

Anatomic diagnosis	Necropsy examination of injection model rabbits 9 months postinjection			Necropsy examination of injury-injection model rabbits 18 months postinjection	
	**R-1**	**R-2**	**R-3**	**R-4**	**R-5**
**Adrenal glands**	Unremarkable	Unremarkable	Unremarkable	Unremarkable	Unremarkable
**Bone marrow**	Unremarkable	Unremarkable	Unremarkable	Unremarkable	Unremarkable
**Brain**	Unremarkable	Unremarkable	Unremarkable	Unremarkable	Unremarkable
**Gall bladder**	Unremarkable	Unremarkable	Unremarkable	Unremarkable	Unremarkable
**Heart**	Unremarkable	Unremarkable	Unremarkable	Unremarkable	Unremarkable
**Kidneys**	Unremarkable similar in size and shape	Unremarkable similar in size and shape	Unremarkable similar in size and shape	Unremarkable, similar in size and shape	Unremarkable, similar in size and shape
**Liver**	Unremarkable	Unremarkable	Unremarkable	Unremarkable	Unremarkable
**Lungs**	Unremarkable	Unremarkable	Unremarkable	Unremarkable	Unremarkable
**Optic nerves**	Unremarkable	Unremarkable	Unremarkable	Unremarkable	Unremarkable
**Pituitary**	Unremarkable	Unremarkable	Unremarkable	Unremarkable	Unremarkable
**Spleen**	Unremarkable	Unremarkable	Unremarkable	Unremarkable	Unremarkable
**Urinary bladder**	Unremarkable	Unremarkable	Unremarkable	Unremarkable	Unremarkable

Note: The three rabbits (R-1, R-2, and R-3) represent the injection model (hESC-derived CECs injected immediately after the removal of the central corneal endothelium (CE)), and the two rabbits (R-4, and R-5) represent the injury-injection model (hESC-derived CECs injected four days after the removal of the central CE).

Supplementary Table 2: The summary of the necropsy examination of monkeys injected with cryopreserved human embryonic stem cell (hESC)-derived corneal endothelial cells (CECs)

Anatomic diagnosis	Necropsy examination of injection model monkeys ≥12 months postinjection			Necropsy examination of injury-injection model monkeys 21 months postinjection	
	**M-1**	**M-2**	**M-3**	**M-4**	**M-5****
**Adrenal glands**	Unremarkable	Unremarkable	Unremarkable	Unremarkable	Unremarkable
**Bone marrow**	Unremarkable	Unremarkable	Unremarkable	Unremarkable	Unremarkable
**Brain**	Unremarkable	Unremarkable	Unremarkable	Unremarkable	Unremarkable
**Gall bladder**	Unremarkable	Unremarkable	Unremarkable	Unremarkable	Unremarkable
**Heart**	Unremarkable	Unremarkable	Unremarkable	Unremarkable	Unremarkable
**Kidneys**	Unremarkable, similar in size and shape	Unremarkable, similar in size and shape	Unremarkable, similar in size and shape	Unremarkable, similar in size and shape	Unremarkable, similar in size and shape
**Liver**	Unremarkable	Unremarkable	Unremarkable	Unremarkable	Unremarkable
**Lungs**	Unremarkable	Unremarkable	Unremarkable	Unremarkable	Unremarkable
**Optic nerves**	Unremarkable, smooth, and symmetric	Unremarkable, smooth, and symmetric	Unremarkable, smooth, and symmetric	Unremarkable, smooth, and symmetric	Unremarkable, smooth, and symmetric
**Pituitary**	Unremarkable	Unremarkable	Unremarkable	Unremarkable	Unremarkable
**Spleen**	Unremarkable	Unremarkable	Unremarkable	Unremarkable	Unremarkable
**Urinary bladder**	Unremarkable	Unremarkable	Unremarkable	Unremarkable	Unremarkable

Note: The monkeys, M-1, M-2, and M-3 represent the injection model (hESC-derived CECs injected immediately after the removal of the central corneal endothelium (CE)), and the monkeys M-4, and M-5 represent the injury-injection model (hESC-derived CECs injected two days after the removal of the central CE). The injection model monkeys (M1 & M2) were housed for 12 months except for M3 that was housed for 26 months postinjection before euthanization and subsequent necropsy examination. **M-5 exhibited the clinical signs of bloody mucoid diarrhea at the euthanization time point and fecal PCR was positive for Shigella and Campylobacter. Importantly, other significant findings were not identified. An examination before euthanization confirmed that the clinical characteristics of the M-3, M-4, and M-5 were in line with the results presented in figures 5, and 6 i.e., the cornea of the injected (right) eye is transparent, and the central corneal thickness of the injected (right) eye remained comparable (± 20 μm) to the untreated (left) eye.

Supplementary Data 1: A complete list of genes identified in non-cryopreserved human embryonic stem cell-derived corneal endothelial cells

The gene expression data were normalized by calculating the FPKM (Fragments per kilobase per million mapped reads) for each gene. A cut-off value of ≥ 1 FPKM was established as an expression threshold for each gene.

Supplementary Data 2: A complete list of genes identified in cryopreserved human embryonic stem cell-derived corneal endothelial cells

The gene expression data were normalized by calculating the FPKM (Fragments per kilobase per million mapped reads) for each gene. A cut-off value of ≥ 1 FPKM was established as an expression threshold for each gene.

Experimental Procedures

Characterization of H9 human embryonic stem cell (hESC)-derived corneal endothelial cells (CECs) by phase-contrast microscopy and quantitative real-time PCR (qRT-PCR)

Phase-contrast microscopy was performed using a Zeiss inverted microscope (Zeiss, Germany), equipped with Q-Capture imaging software (QImaging, Surrey, BC, Canada). Total RNA for qRT-PCR from H9 hESCs, hESC-derived CECs at day 20, and cryopreserved hESC-derived CECs was extracted using TRIzol reagent (Invitrogen; Carlsbad, CA). First-strand cDNA synthesis was completed using the Superscript III kit (Invitrogen) according to the manufacturer's instructions.

The expression of corneal endothelium (CE)-associated markers (*AQP1, ATP1A1, TJP1, COL4A1, COL4A3, COL8A1, COL8A2, FOXC1, and SLC16A3*) were quantitated using qRT-PCR as described (Ali et al., 2018). The primers were designed using a real-time PCR tool (Integrated DNA Technologies; Coralville, IA) and are available upon request. qRT-PCR was performed on STEP ONE ABI Real-Time PCR System (Applied Biosystems; Foster City, CA). Delta-delta C_T method was used to determine the relative expression, normalized against *GAPDH* expression (Livak and Schmittgen, 2001).

Characterization of hESC-derived CECs by immunocytochemistry

The hESC-derived CECs were examined by immunocytochemistry. Briefly, the cells were fixed with 4% paraformaldehyde (PFA) for 15 minutes followed by blocking with 5% bovine serum albumin (MilliporeSigma; Burlington, MA). The cells were first incubated with 1:100 zona occludens-1 (ZO-1; catalog # 13663s; Cell Signaling Technology; Danvers, MA) primary antibody overnight at 4°C. The cells were next treated with 1:100 FITC-conjugated goat anti-rabbit IgG (catalog # AP307F; MilliporeSigma) secondary antibody for 2 hours at room temperature. The nuclei were counterstained with DAPI (4′,6-diamidine-2′-phenylindole dihydrochloride; MilliporeSigma). The images of mounted cells were captured using an Olympus

LX81 microscope (Olympus, Tokyo, Japan) equipped with software (Slidebook Software 3i; Denver, CO) and prepared using image-editing software (Adobe Photoshop CS5; Adobe Systems, Inc., San Jose, CA).

Next-generation RNA sequencing (RNA-Seq) of hESC-derived CECs

Next-generation RNA-Seq of hESC-derived CECs at day 20 (non-cryopreserved) and cryopreserved hESC-derived CECs (cryopreserved for 40 days in liquid nitrogen) was performed. Briefly, three biological replicates for each of non-cryopreserved hESC-derived CECs and cryopreserved hESC-derived CECs were used for RNA-Seq library preparation. Total RNA was isolated from each sample using TRIzol reagent (Invitrogen). The extracted RNA was examined using a NanoDrop Lite spectrophotometer (Thermo Fisher Scientific) and RNA 6000 Pico kit on an Agilent 2100 Bioanalyzer (Agilent; Palo Alto, CA). RNA-Seq library preparation, next-generation sequencing, and analysis of the RNA-Seq datasets including reads mapping to reference genome, quantification of gene expression, differential gene expression analysis, and correlations were performed commercially by Novogene Corporation Inc (Sacramento, CA). All raw sequencing data reported in this manuscript have been deposited in NCBI's Gene Expression Omnibus (Edgar et al., 2002).

References

Ali, M., Khan, S.Y., Vasanth, S., Ahmed, M.R., Chen, R., Na, C.H., Thomson, J.J., Qiu, C., Gottsch, J.D., and Riazuddin, S.A. (2018). Generation and Proteome Profiling of PBMC-Originated, iPSC-Derived Corneal Endothelial Cells. Invest Ophthalmol. Vis. Sci. 59, 2437-2444.

Edgar, R., Domrachev, M., and Lash, A.E. (2002). Gene Expression Omnibus: NCBI gene expression and hybridization array data repository. Nucleic Acids Res. 30, 207-210.

Livak, K.J., and Schmittgen, T.D. (2001). Analysis of relative gene expression data using real-time quantitative PCR and the 2(-Delta Delta C(T)) Method. Methods 25, 402-408.

Mortazavi, A., Williams, B.A., McCue, K., Schaeffer, L., and Wold, B. (2008). Mapping and quantifying mammalian transcriptomes by RNA-Seq. Nat. Methods 5, 621-628.
